# Evaluation of Robustness of S-Transform Based Phase Velocity Estimation in Viscoelastic Phantoms and Renal Transplants

**DOI:** 10.1109/TBME.2023.3323983

**Published:** 2024-02-26

**Authors:** Piotr Kijanka, Luiz Vasconcelos, Jay Mandrekar, Matthew W. Urban

**Affiliations:** Department of Robotics and Mechatronics, AGH University of Krakow, 30-059 Krakow, Poland; Department of Radiology, Mayo Clinic, USA.; Department of Quantitative Health Sciences, Division of Clinical Trials and Biostatistics, Mayo Clinic, USA.; Department of Radiology, Mayo Clinic, USA, and also with the Department of Physiology and Biomedical Engineering, Mayo Clinic, USA.

**Keywords:** Dispersion, acoustic radiation force (ARF), in vivo, kelvin-voigt, kidney, phantom, shear wave elastography (SWE), stockwell transform, ultrasound, viscoelasticity, zener

## Abstract

Ultrasound shear wave elastography (SWE) methods are being used to differentiate healthy versus diseased tissue on the basis of their viscoelastic mechanical properties. Tissue viscoelasticity is often studied by analyzing shear wave phase velocity dispersion curves, which is the variation of phase velocity with frequency or wavelength. Recently, a unique approach using a generalized Stockwell transformation (GST-SFK) was proposed for the calculation of dispersion curves in viscoelastic media over expanded frequency band. In this work, the method’s robustness was evaluated on data from five custom-made viscoelastic tissue-mimicking phantoms and sixty in vivo renal transplants. For each phantom, 15 shear wave motion data acquisitions were taken, while 10–13 acquisitions were acquired for renal transplants measured in the renal cortex. For each data-set mean and standard deviation (SD) of estimated phase velocity dispersion curves were studied. In addition, the viscoelastic parameters of the Zener model were examined, which were preceded by a convergence analysis. For viscoelastic phantoms scanned with a research ultrasound scanner, and for the in vivo renal transplants scanned with a clinical scanner, the decisive advantage of the GST-SFK method over the standard two-dimensional Fourier transform (2D-FT) method was shown. The GST-SFK method provided dispersion curve estimates with lower SD over a wider frequency band in comparison to the 2D-FT method. These advantages are relevant to the analysis of the mechanical properties of tissues in clinical practice to discriminate healthy from diseased tissue.

## I. Introduction

Shear wave elastography (SWE) was proposed more than two decades ago for the non-invasive evaluation of the mechanical properties of tissues [1], [2]. Since then, SWE has proven to be a useful method for quantifying the viscoelastic properties of different types of tissue, including liver, kidney, skeletal muscle, prostate, and heart [3], [4], [5], [6]. SWE methods have emerged as a way to distinguish healthy from diseased tissue based on the viscoelastic properties of the tissue. Typically, SWE techniques use focused ultrasound beams to produce an acoustic radiation force (ARF) excitation to generate propagating shear waves [7], [8]. The shear wave motion is measured using ultrafast ultrasound imaging techniques. Then, shear wave velocity in the time- or frequency-domain is estimated using various techniques, which is related to the mechanical properties of the tissue.

In the case of viscoelastic media, the wave velocity changes with frequency, which is called dispersion [9]. Dispersion curves are plots of phase velocities, or equivalent wavenumbers, as a function of frequency and are related to the elasticity and viscosity of the tissue [10]. The dispersion curves are often fit with relationships described by rheological models (e.g., Zener (Standard Linear Solid), Kelvin-Voigt, Maxwell, Kelvin-Voigt fractional derivative) for better tissue characterization [11], [12], [13], [14], [15]. Nightingale, et al., noted that there are large differences in Kelvin-Voigt (KV) fitting parameters for limited frequency ranges, in particular for highly dispersive media [15]. These are significant limitations in employing the KV, and potentially other rheological models for the frequency domain analysis. Therefore, attention is paid to estimate robust dispersion curves in viscoelastic materials and tissues over an extended frequency range, which in turn will improve the use of rheological models for more accurate tissue characterization.

To date, many techniques for phase velocity dispersion curve calculation have been proposed. The most known, and widely used due to its simplicity, is a two-dimensional Fourier transform (2D-FT) [16], [17]. The 2D-FT approach of spatiotemporal motion data creates the frequency-wavenumber (f-k) distribution, also known as k-space, that shows the distribution of shear wave magnitude. The peaks of the f-k distribution represent the phase velocities of different wave propagation modes as (f)=2*π*f/k=2*π*f*λ. Another commonly used technique uses a phase gradient to calculate the phase velocity [18]. In this method, shear wave phases are measured at each frequency of interest for all spatial locations, and a linear regression or curve fit is used to calculate the phase velocity. The phase gradient and 2D-FT techniques cover the vast majority of research related to the analysis of dispersion curves in elastic and viscoelastic soft materials and tissues. Nevertheless, it was shown that more advanced methods can offer better frequency resolution, and lower variance in the presence of noise in the measured data [19]. There are many other techniques for estimating dispersion relations, including non-parametric, parametric and high-resolution methods, which can offer better accuracy [15], [19], [20], [21]. Among them, a Radon sum, Multiple Signal Classification (MUSIC), eigenvector, Blackman-Tukey have been reported.

Recently, a unique approach for shear wave dispersion curves calculation was proposed for use in viscoelastic materials and tissues [22]. The proposed method, named GST-SFK, uses a generalized form of the Stockwell (S-transform) transform along with the slant f-k analysis. It uses the S-transform that combines strengths of the short time FT, and the continuous wavelet transform method to overcome their weaknesses. The method was investigated on numerical and experimental phantom data, and also a limited number of ex vivo and in vivo experimental tissue liver data using single data acquisitions [22]. The authors have shown the promise of the GST-SFK method for phase velocity estimation with expanded usable bandwidth by a factor of two or more in viscoelastic phantoms and tissues. The GST-SFK approach achieved much better performance compared to the 2D-FT and high-resolution (eigenvector) methods, making it very competitive for clinical applications [22].

In this work, we further explore the use of the GST-SFK approach for phase velocity estimation in viscoelastic phantoms and more challenging clinical applications, i.e. renal transplants. A renal biopsy is the gold standard in kidney health diagnosis, but it is an invasive procedure, cannot be used frequently, and can cause complications [23]. Noninvasive SWE techniques are being studied to discriminate healthy versus diseased renal tissue [24],[25],[26],[27],[28]. Renal inflammation is the initial response to renal injury. Prolonged inflammation promotes the process of fibrosis, leading to chronic kidney rejection. The GST-SFK approach can potentially help provide a more accurate assessment of renal health via providing robust phase velocity dispersion curves over extended frequency band.

This article is organized in the following way. First, we briefly recall the theory for the GST-SFK method proposed in [22], along with the commonly used 2D-FT approach. These methods were tested on particle velocity data from five custom-made tissue-mimicking (TM) viscoelastic phantoms and sixty in vivo renal transplants. The robustness of the methods was tested by analyzing the responses for 15 and 10–13 shear wave data acquisitions at various spatial locations for the phantoms and renal transplants, respectively. The results will be followed by a discussion and conclusions.

## II. Methods and Materials Description

### A. S-Transform-Based Method (GST-SFK)

A generalized Stockwell transformation combined with a slant f-k analysis was proposed for shear wave phase velocity estimation in [22]. The GST-SFK method uses a generalized S-transform for a time-frequency decomposition of a signal with a frequency-dependent Gaussian window. The generalized S-transform can be described as

(1) $$S\left[v\left(\tau \right)\right]\left(\tau ,f,\beta \right)=\int_{-\infty }^{+\infty }\;v\left(t\right)\left[\frac{\left|f\right|}{\sqrt{2\pi \beta }}e^{-\frac{f^{2}{\left(\tau -t\right)}^{2}}{2\beta }}\right]e^{-i2\pi ft}dt,$$

where the time-frequency resolution is controlled by the β scaling factor, which changes the width of the scaled Gaussian window. The function S indicates the time-frequency S-transform of the time variable v(t) signal, f is a frequency and τ is responsible for controlling the position of the Gaussian window in the time domain, t. For a narrow window in the time domain, the S-transform resolution in the frequency domain decreases, and inversely, widening the Gaussian window in the time domain increases the frequency resolution.

A shear wave wave field, v(x,t), is transformed to the time-frequency using (1). Then, a series of 2D complex-valued functions of the time and distance is calculated as

(2) $$V\left(x,\tau \right)=S\left[v\left(x,t\right)\right]\left(\tau ,f,x\right).$$

Next, the one-dimensional complex-valued slant-phase function, P, is considered. The P function in a form

(3) $$P\left(x\right)=V\left(x,\frac{x}{u_{m}}=\tau \right)$$

is considered for a selected frequency, steering group velocity u=x/τ, and the constant time. It is computed for a number of steering group velocity values $u_{m}$, a maximum distance $x_{m}$, and a maximum time $t_{m}$, i.e. $u_{m}=\frac{x_{m}}{t_{m}-m\Delta t}$ with Δt being the time sampling rate. Following, the slant-phase function amplitude is calculated as

(4) $$\Lambda \left(u,f,k\right)=\left|\int_{-\infty }^{+\infty }\;\;P\left(x\right)e^{-2i\pi kx}dx\right|.$$

The Λ parameter is a spectral amplitude distribution with the steering group velocity, frequency, and wavenumber coordinates, respectively. A maximum amplitude of Λ(u,f,k) over all steering group velocities is taken for the dispersion curves calculation, which can be described as

(5) $$K\left(f,k\right)=\underset{u}{max}\;\left[\Lambda \left(u,f,k\right)\right].$$

Then, phase velocity dispersion curves were computed from finding the peaks in the K(f,k) distribution. It should be noted that in the GST-SFK method each frequency is treated independently and no extrapolation process is used. The GST-SFK method utilizes the slant f-k transform and the generalized S-transform to convert data from the time-space domain to the frequency-phase velocity domain, effectively mitigating noise at each frequency and reducing spatial spectral leakage artifacts [22].

### B. Two-Dimensional Fourier Transform (2D-FT)

The two-dimensional Fourier transform (2D-FT) is the most used method for phase velocity dispersion curve calculation in biomedical ultrasound. It belongs to a class of non-parametric approaches which are based on the idea of estimating the autocorrelation sequence of a random process from a set of measured data [19].

The 2D-FT is performed in the temporal and spatial domains to obtain an estimate of the frequency-wavenumber, f-k, distribution. Next, the f-k distribution is converted to phase velocity curves which can be done in two different ways, from finding the peaks in the f-k distribution along a given search direction, or zero-crossing locations after applying a gradient operator [20]. The coordinates of the peaks or zero-crossing locations are then used to calculate the phase velocity as c(f)=2πf/k.

In this work, maximum peaks from the f-k distribution were detected for each frequency. To extract the main shear wave mode from the detected peaks, the peaks corresponding to the shear wave mode were tracked for the closest value. Then, the coordinates of the localized peaks were used to calculate the phase velocity c(f).

### C. Tissue-Mimicking Viscoelastic Phantoms

Five viscoelastic, custom-made, tissue-mimicking (TM) phantoms (CIRS, Inc., Norfolk, VA, USA, manufactured in 2017–2018) were used for the purposes of determining shear wave phase velocity estimation robustness, using the S-transform-based method. They are denoted as phantoms with Roman numerals I-V for this article. The reference mechanical properties of these phantoms are unknown. Shear wave acquisitions were performed with a Verasonics system (V1, Verasonics, Inc., Kirkland, WA, USA), and a linear array transducer (L7–4, Philips Healthcare, Andover, MA). Data were taken for each phantom, for different spatial positions, corresponding to 15 data acquisitions, to explore biases related to dispersion curves calculation. The ARF push beams were generated and focused at 21.56 mm in depth. The push duration, and the push frequency were 400 *μ*s and 4.09 MHz, respectively. A plane wave acquisition, using three plane waves that were coherently compounded (−4°, 0°, +4°), was used [29]. The effective frame rate after compounding was 4.167 kHz. The shear wave particle velocity motion data were calculated from the in-phase/quadrature (IQ) data using an autocorrelation algorithm [30]. The particle velocity signals, shown in a Supplementary Material, were measured in the lateral segment length starting from 0 to 30 mm, and were averaged over a range of 5 mm in the axial direction centered at the focal depth, before starting to determine the dispersion curves.

### D. in vivo Renal Transplant Data

Sixty in vivo renal transplant subjects were also used to test the robustness of the GST-SFK method, for clinical use. Shear wave measurements were conducted on human subjects scheduled to undergo protocol biopsy. The kidney imaging and measurements were carried out prior to the biopsy under a protocol approved by the Mayo Clinic Institutional Review Board. Written informed consent was obtained prior to scanning. The examinations were carried out by an experienced clinical sonographer. During the tests, the ultrasound probe was positioned to find a longitudinal plane of the kidney, and the region-of-interest (ROI) was positioned in the middle of the kidney to make measurements in the renal cortex. Data acquisition was performed using a Logiq E9 ultrasound system equipped with C1–6-D curved array transducer (General Electric Company, Wauwatosa, WI, USA). Up to 10–13 acquisitions were taken for each subject. The ARF push beams were focused at the edges of the ROI and directional filtering was used upon the shear wave field to extract the leftward from rightward traveling shear waves. Ultrasound data were taken to measure the shear wave motion with a frame rate of 2.412 kHz. When analyzed, the data were manually selected from the renal cortex based on B-mode images. The particle velocity signals, shown in Supplementary Material, were measured in the lateral segment length starting from 0 to 21.3 mm.

The biopsy tissue histology was analyzed by Mayo Clinic nephropathologists and characterized using the Banff criteria [31], [32]. We noted the presence of inflammation, interstitial fibrosis, and tubular atrophy. In this data set, there were cases of mild to moderate disease in the aforementioned categories.

Subjects were divided into four groups: A - healthy subjects; B - subjects with inflammation and no interstitial fibrosis and tubular atrophy (IFTA); C - subjects with IFTA, but no inflammation; and D - subjects with IFTA and inflammation. All four groups are summarized in Table I. All groups consisted of 15 subjects each. For each subject group, the corresponding values of group velocities are given, which were estimated using a space-time thresholded motion search [33].

### E. Zener Rheological Model

Based on the phase velocity estimates over a given frequency range, the elasticity and viscosity parameters were deduced. In this work, we used the Zener rheological viscoelastic model. The Zener model is composed of a dashpot (viscosity, η) and two spring elements (Young’s moduli, $E_{1}$ and $E_{2}$). The stress-strain relationship of the Zener model is given in the form [34]

(6) $$\sigma \left(t\right)+\tau_{\sigma }\frac{\partial \sigma \left(t\right)}{\partial t}=E_{1}\left[\varepsilon \left(t\right)+\tau_{e}\frac{\partial \varepsilon \left(t\right)}{\partial t}\right],$$

where the relaxation time $\tau_{\sigma }=\eta /E_{2}$, the retardation time $\tau_{e}=\eta /E_{1}+\eta /E_{2}$, and $\tau_{\sigma }⩽\tau_{e}$. Solving (6) leads to the frequency-dependent phase velocity of the Zener model, which can be written as

(7) $$V_{s}\left(\omega \right)=\sqrt{\frac{2}{3\rho }\frac{A^{2}\;+\;B^{2}}{\left(E_{2}^{2}\;+\;\eta^{2}\omega^{2}\right)(B\;+\;\sqrt{A^{2}+\;B^{2}})}},$$

where $A=\eta \omega E_{2}^{2},\;B=E_{1}E_{2}^{2}+\eta^{2}\omega^{2}\left(E_{1}+E_{2}\right).\;\rho$ is the density and ω is an angular frequency, i.e., ω=2πf. When $E_{2}$ reaches a very high level, the Zener model begins to behave like the Kelvin-Voigt viscoelastic model.

In order to estimate $E_{1},\;E_{2}$ and η parameters, $V_{s}(\omega )$ was estimated using a nonlinear least-squares problem (NLSQ) in a form

(8) $$\left[E_{1},E_{2},\eta \right]=\underset{E_{1},E_{2},\eta }{min}\;{∥V_{s}(f)-V_{ph}(f)∥}_{2}^{2}.$$

Equation (8) was numerically solved using the MATLAB solver *lsqcurvefit*. The Zener fit was done using two sets of frequency ranges: a short one (with reduced variability and SD) that one would select based on the phase velocity curves estimated using the 2D-FT method, and an extended frequency range. The short frequency range used for the KV fit and the TM phantoms was: 150–400 Hz for Phantom I, 150–1400 Hz for Phantom II, 150–1400 Hz for Phantom III, 150–700 Hz for Phantom IV, and 150–600 Hz for Phantom V. The extended frequency range of 150–1800 Hz for the TM phantoms was used.

Three frequency ranges were used for renal transplants, i.e.:

- *Case 1:* a fixed frequency range of 200–450 Hz, where all groups (except D for 2D-FT) had a coefficient of variation (CV) < 30%;
- *Case 2:* a fixed frequency range of 200–900 Hz;
- *Case 3:* frequency range starting from 200 Hz up to the maximum frequency for which CV < 30% for a given subject group and given approach.

Coefficient of variation, defined as $CV=\frac{SD}{MEAN}\cdot 100\%$ shows the degree of variation relative to the sample mean. The lower the CV, the smaller the variation. For each curve fit the norm of residuals (NoR) was measured. The norm of residuals, i.e., the squared L_2_-norm of the residual, is a measure of the goodness of Zener fit, where a smaller value indicates a better fit than a larger value [35].

### F. Statistical Tests

In this study there were three factors of interest: group (4 levels, Groups A, B, C, D), method (2 levels, 2D-FT and GST-SFK) and frequency (24 levels). Analysis was performed using three-way fixed effect ANOVA with two factors repeated (method and frequency). Each group had 15 subjects (i.e., a total of 60 subjects). Multiple observations within each combination of group by method by frequency for each patient were summarized as mean ± standard deviation. These means of the phase velocity at each level (i.e., a total of 2880 = 15 subjects × 4 groups × 2 methods × 24 frequencies) were used as an outcome variable in this analysis. Given that we have three factors, we used Mauchly’s sphericity test to decide on the appropriateness of a multivariable approach. A statistically significant *p*-value (*p* < 0.05) for this test indicates that correlations between each pair of repeated measures are not the same. An assessment of statistical significance for each of the main effects and the two-factor and three-factor interaction terms (group by method, group by frequency, method by frequency and group by method by frequency) were assessed using a test based on Pillai’s trace.

For each of the methods, the extent of variability relative to mean was assessed using the coefficient of variation, which is the ratio of standard deviation to mean. This was done separately within each group and for all frequencies. We compared estimated $E_{1},\;E_{2}$, and η parameters for Cases 1, 2, and 3 as described below. We used a Wilcoxon rank sum test for pairwise comparison to determine if there were differences between the median values of the subject groups. Specifically, we investigated if there were any significant differences in the results when comparing healthy subjects (group A) with other groups (B, C, D) in a pairwise manner.

## III. Results

### A. TM Phantoms

**Fig. 1.**
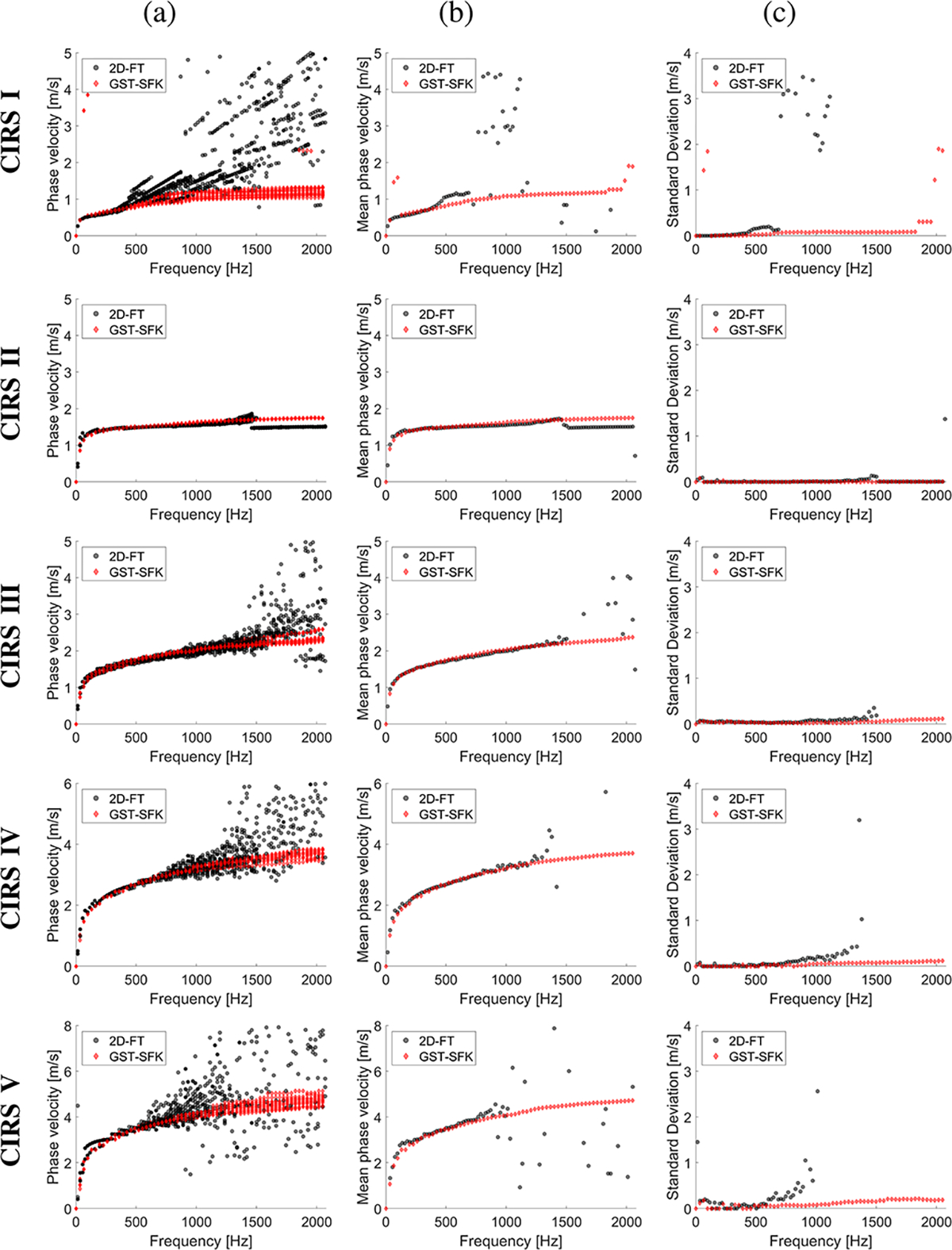
Phase velocity curves calculated for the 2D-FT (black dots), and GST-SFK (red diamonds) methods. Results were computed for the CIRS tissue-mimicking viscoelastic phantoms, for (a) 15 data acquisitions. (b) mean, and (c) standard deviation (SD) were calculated on the basis of all acquisitions from (a).

Shear wave particle velocity motion data for custom-made TM viscoelastic phantoms were examined. Shear wave spatiotemporal data and the frequency-wavenumber (f-k) distribution, also known as *k*-space which shows the distribution of shear wave energy, reconstructed based on the 2D-FT, and GST-SFK methods were shown in a Supplementary Material in Fig. S1. Phase velocity curves calculated for all the TM viscoelastic phantoms, and 15 data acquisitions acquired for each phantom, were plotted in Fig. 1(a). Based on all data acquisitions collected for various spatial locations, mean and standard deviation (SD) curves were calculated in the frequency domain and plotted in Fig. 1(b) and (c).

Dispersion curves computed for the softest phantom, Phantom I, and for all fifteen data acquisitions have high variation starting from 500 Hz, for the 2D-FT method (the top row in Fig. 1(a)). This results in increased mean phase velocity which was also enlarged with increasing frequency. This variation was presented as standard deviation in Fig. 1(c), where SD values slightly increase up to approximately 0.18 m/s in the frequency range from 400 to 660 Hz, and then drastically jumps to 2 m/s and above. The GST-SFK approach on the other hand, provides much more stable dispersion curves between all the acquisitions, up to almost 1900 Hz, where SD values do not exceed 0.08 m/s.

The 2D-FT approach, for Phantom II, provided robust estimates up to approximately 1400 Hz with low SD values at the level of 0.06 m/s. Then, above 1400 Hz mean phase velocity for 2D-FT was discontinued and underestimated, besides having small SD (< 0.01 m/s). In turn, the GST-SFK method estimated robust phase velocity curves for all frequency band with SD values being lower than 0.01 m/s.

Measurements carried out on Phantoms III, IV and V also revealed the advantage of the GST-SFK method over the 2D-FT technique. It can be seen that phase velocity has higher variability as the frequency increases, between different data acquisitions for 2D-FT. This results in a significant increase in SD, and deviations in the mean phase velocity. The reliable mean phase velocity, calculated using 2D-FT, was up to approximately 1500 Hz, 1200 Hz and 700 Hz, for Phantoms III, IV, and V, respectively. For these frequency ranges, the SD values were at the level below 0.20 m/s. While the GST-SFK approach had estimated stable phase velocity curves over the entire frequency band, with SD values at the level below 0.10 m/s for Phantoms III and IV, and 0.20 m/s, for Phantom V, respectively.

**Fig. 2.**
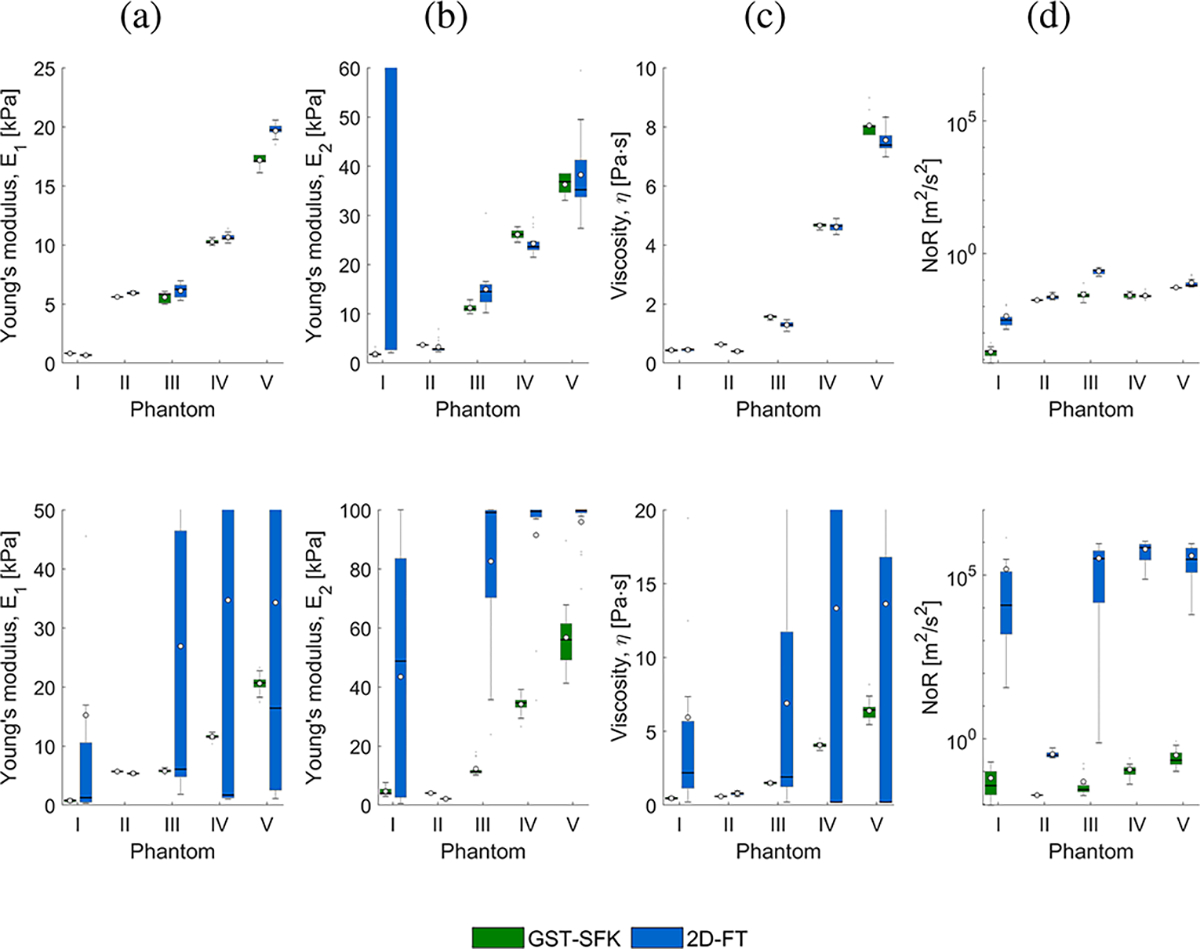
Box plots calculated for estimated Zener parameters Young’s modulus, $E_{1}$ (a), Young’s modulus, $E_{2}$ (b), viscosity, η (c), and the norm of residuals, NoR (d), for GST-SFK and 2D-FT methods. White circles represent mean values, whereas a solid line within the box corresponds to a median value. Results are presented for the TM viscoelastic phantoms I-V. The top row presents results for the Zener fit performed using a short frequency ranges: 150–400 Hz for I, 150–1300 Hz for II, 150–1400 Hz for III, 150–700 Hz for IV, and 150–600 Hz for V. The bottom row shows estimates for the Zener fit done using an extended frequency range from 150 to 1800 Hz for all the phantoms. Note the logarithmic vertical axis for the norm of residuals. (a) $E_{1}$. (b) $E_{2}$. (c) η. (d) NoR.

Fig. 2 shows box plots calculated for estimated Zener parameters $E_{1},\;E_{2}$, and η, and the norm of residuals, NoR, for GST-SFK and 2D-FT methods. Results for five TM phantoms investigated in this work are presented. The bottom and top edges of the box indicate the 25^th^ and 75^th^ percentiles, respectively. The white circles represent the mean values, while the solid line in each box corresponds to a median value of the phase velocity, respectively. Outliers were plotted for values greater than $r_{3}+w_{l}\left(r_{3}-r_{1}\right)$ or less than $r_{1}-w_{l}\left(r_{3}-r_{1}\right)$, where $w_{l}=1.5$ is the maximum whisker length, and $r_{1}$ and $r_{3}$ are the 25^th^ and 75^th^ percentiles of the sample data, respectively.

Box plots for all the phantoms and $E_{1}$ (Fig. 2(a), top row), $E_{2}$ (Fig. 2(b), top row), and η (Fig. 2(c), top row) were at a comparable level for the two methods and the short frequency range, except for the 2D-FT approach and Phantom I, for which the $E_{2}$ had a large boxplot. The NoR values also oscillated at the same level between the 2D-FT and GST-SFK methods (Fig. 2(d)).

For the extended frequency range, box plots with lower variability were estimated for the Zener viscoelastic parameters $E_{1}$ (Fig. 2(a), bottom row), $E_{2}$ (Fig. 2(b), bottom row), and η (Fig. 2(c), bottom row), for the GST-SFK method compared to the 2D-FT technique for Phantoms I and III-V. It was caused by much lower phase velocity variation at higher frequencies for the GST-SFK method, where the norm of residuals was below 0.1 m^2^/s^2^, compared to the 2D-FT method, for which NoR oscillated between 10^4^-10^6^ for Phantoms I and III-V (Fig. 2(d)). The NoR parameter for Phantom II was below a value of 0.1 m^2^/s^2^ for the two methods, which gave robust viscoelastic Zener parameters estimation.

### B. In Vivo Renal Transplants

**Fig. 3.**
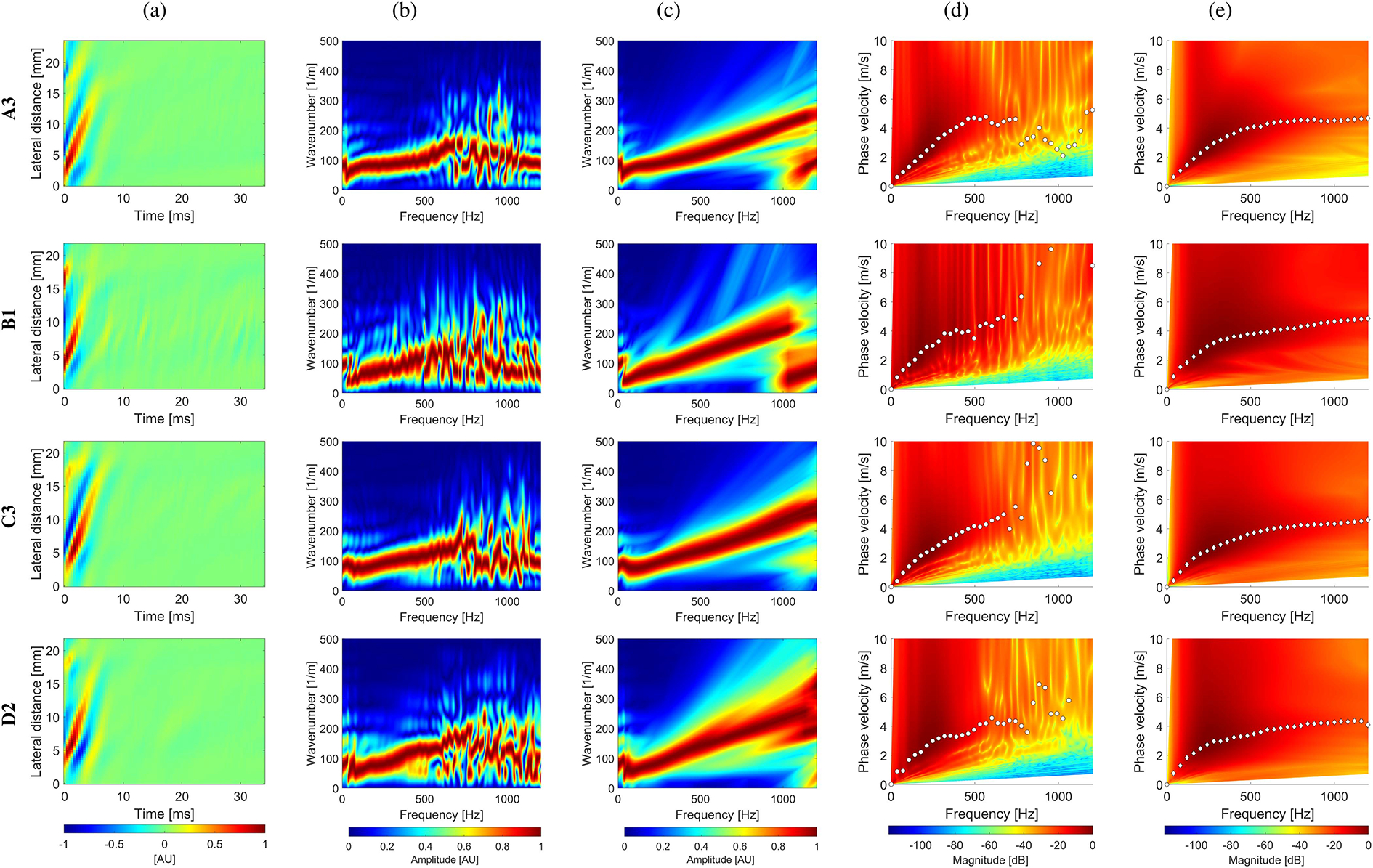
(a) Spatiotemporal shear wave particle velocity signals. The frequency-wavenumber (f-k) distribution reconstructed based on the (b) 2D-FT, and (c) GST-SFK methods. The f-k maps are normalized by wavenumber maxima in the frequency direction. Phase velocity reconstructions based on the (d) 2D-FT, and (e) GST-SFK methods, for shear wave motion measurements. The phase velocity maps have superimposed markers corresponding to the maximum peaks of the phase velocity. Results were calculated for Groups A-D in vivo renal transplants, for randomly selected data acquisitions.

**Fig. 4.**
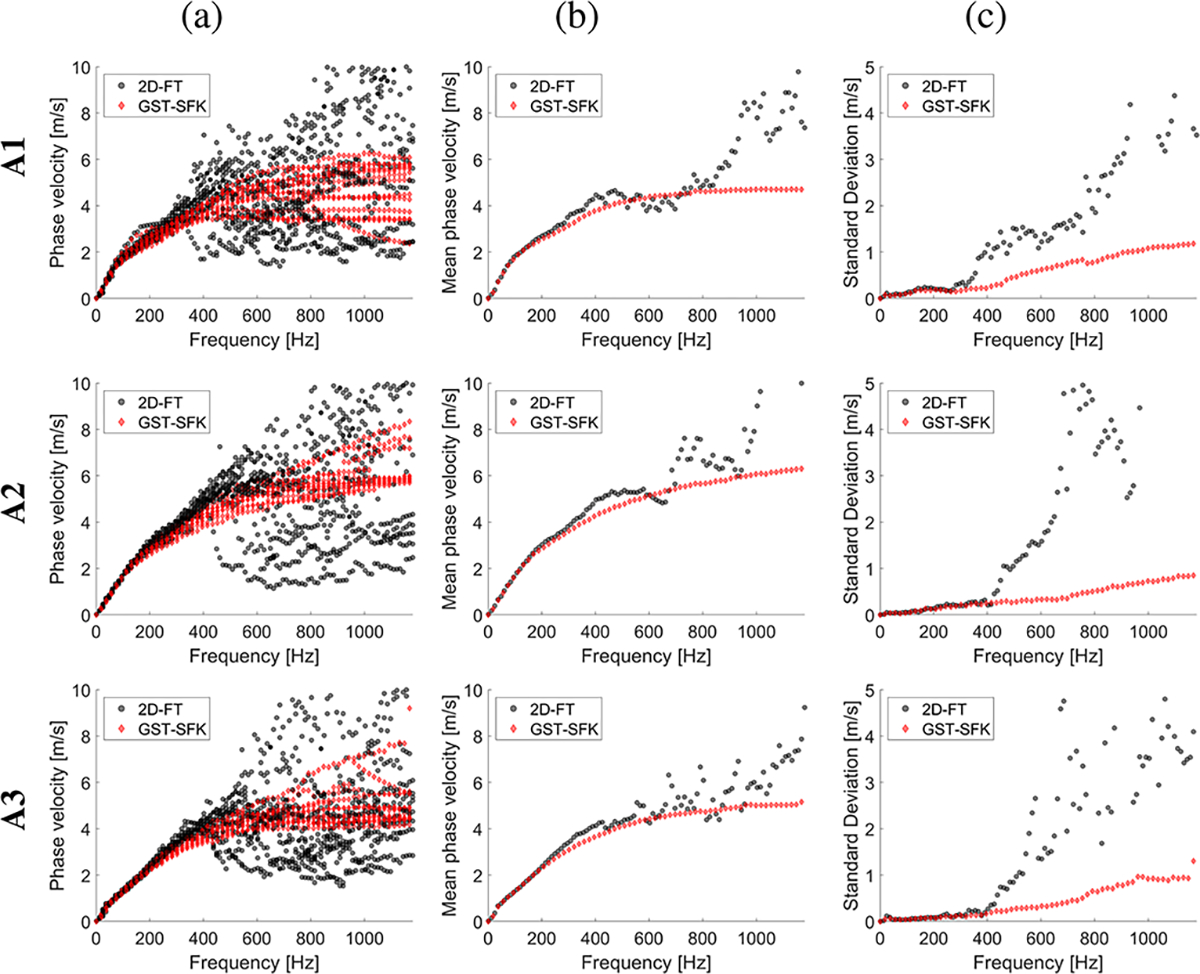
Phase velocity curves calculated for the 2D-FT (black dots), and GST-SFK (red diamonds) methods. Results were computed for the normal (Group A) in vivo renal transplants. Results were calculated for (a) 13 data acquisitions. (b) mean, and (c) standard deviation (SD) were calculated on the basis of all acquisitions from (a).

**Fig. 5.**
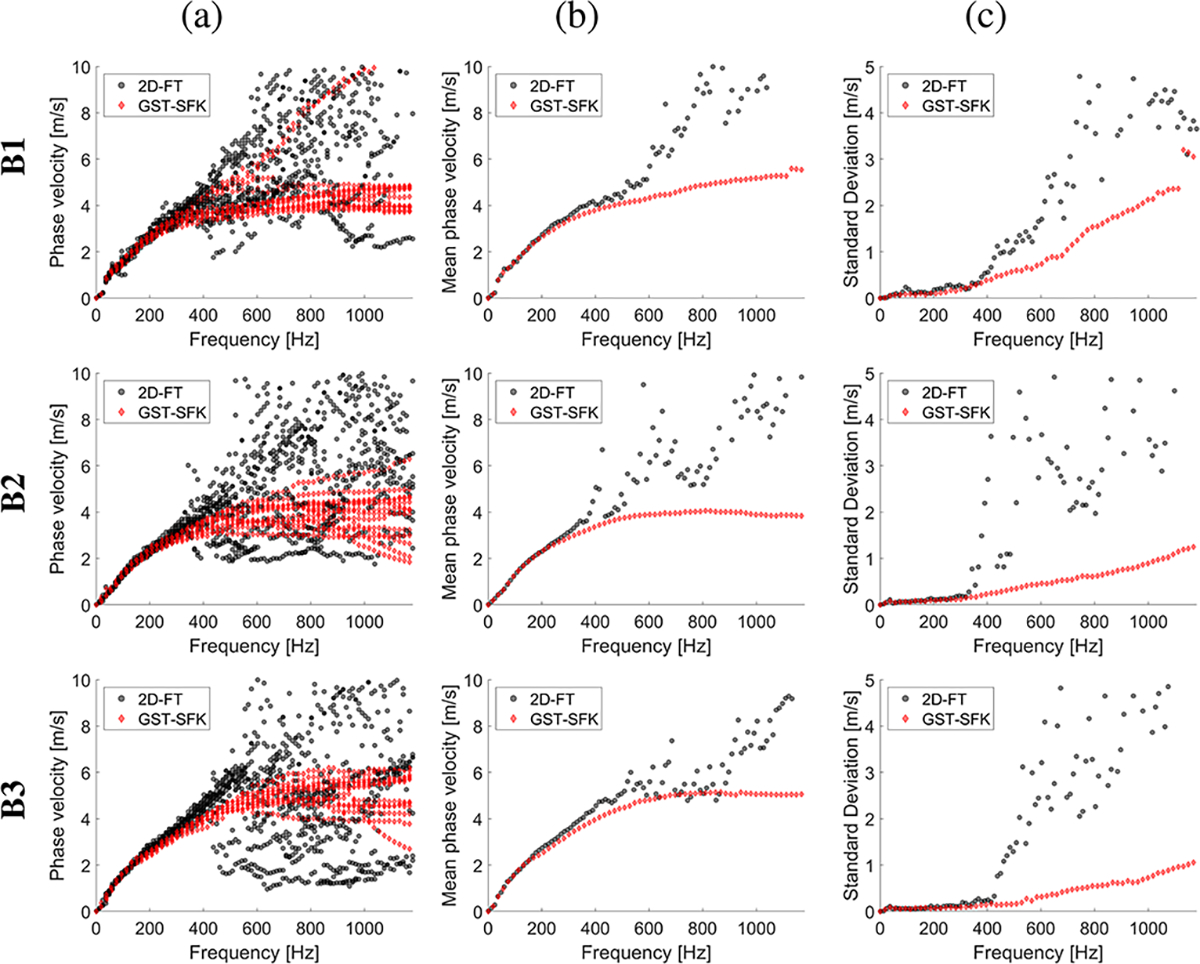
Phase velocity curves calculated for the 2D-FT (black dots), and GST-SFK (red diamonds) methods. Results were computed for the in vivo renal transplants, for subjects with inflammation and no IFTA (Group B). Results were calculated for (a) 13 data acquisitions. (b) mean, and (c) standard deviation (SD) were calculated on the basis of all acquisitions from (a).

**Fig. 6.**
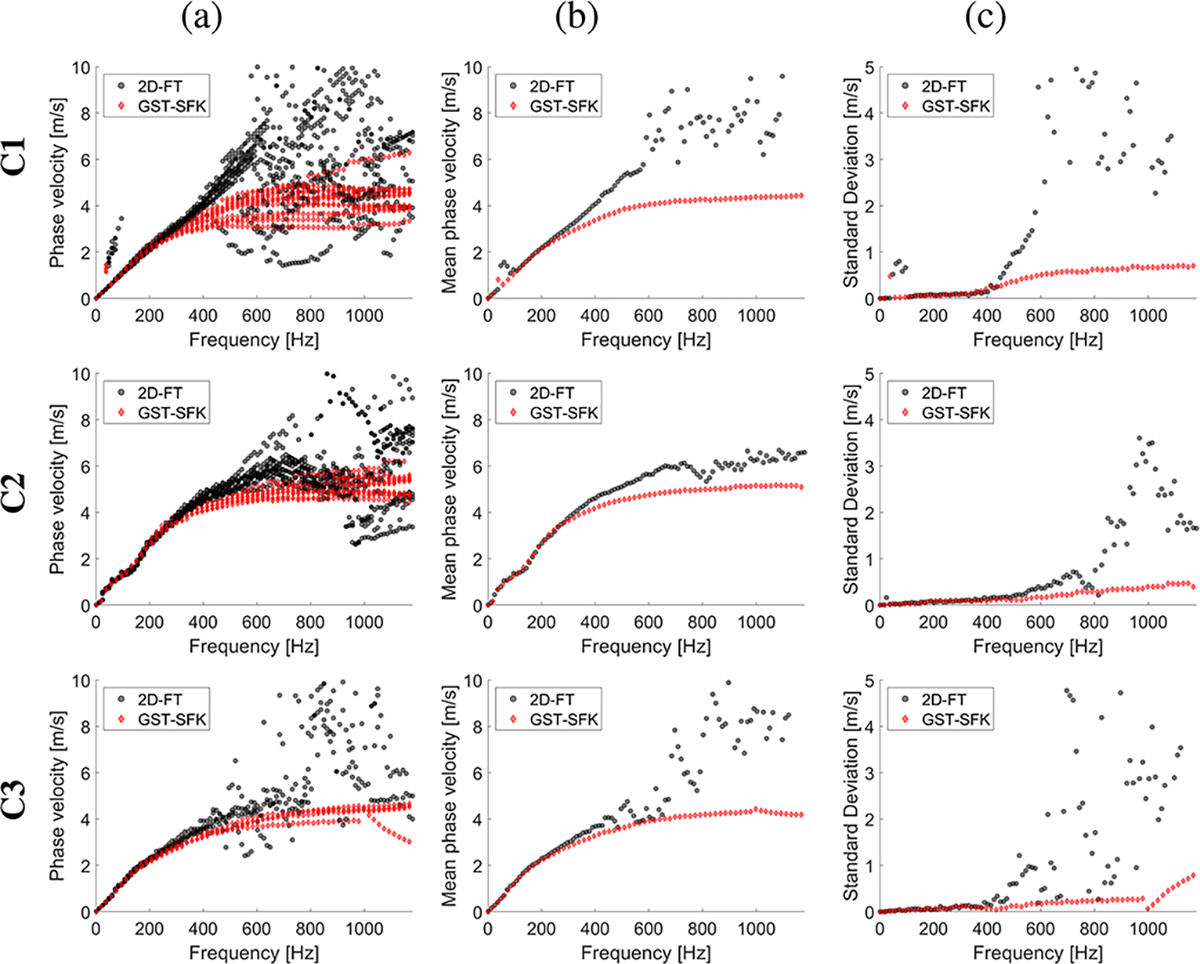
Phase velocity curves calculated for the 2D-FT (black dots), and GST-SFK (red diamonds) methods. Results were computed for the in vivo renal transplants, for subjects with IFTA but no inflammation (Group C). Results were calculated for (a) 13 data acquisitions. (b) mean, and (c) standard deviation (SD) were calculated on the basis of all acquisitions from (a).

**Fig. 7.**
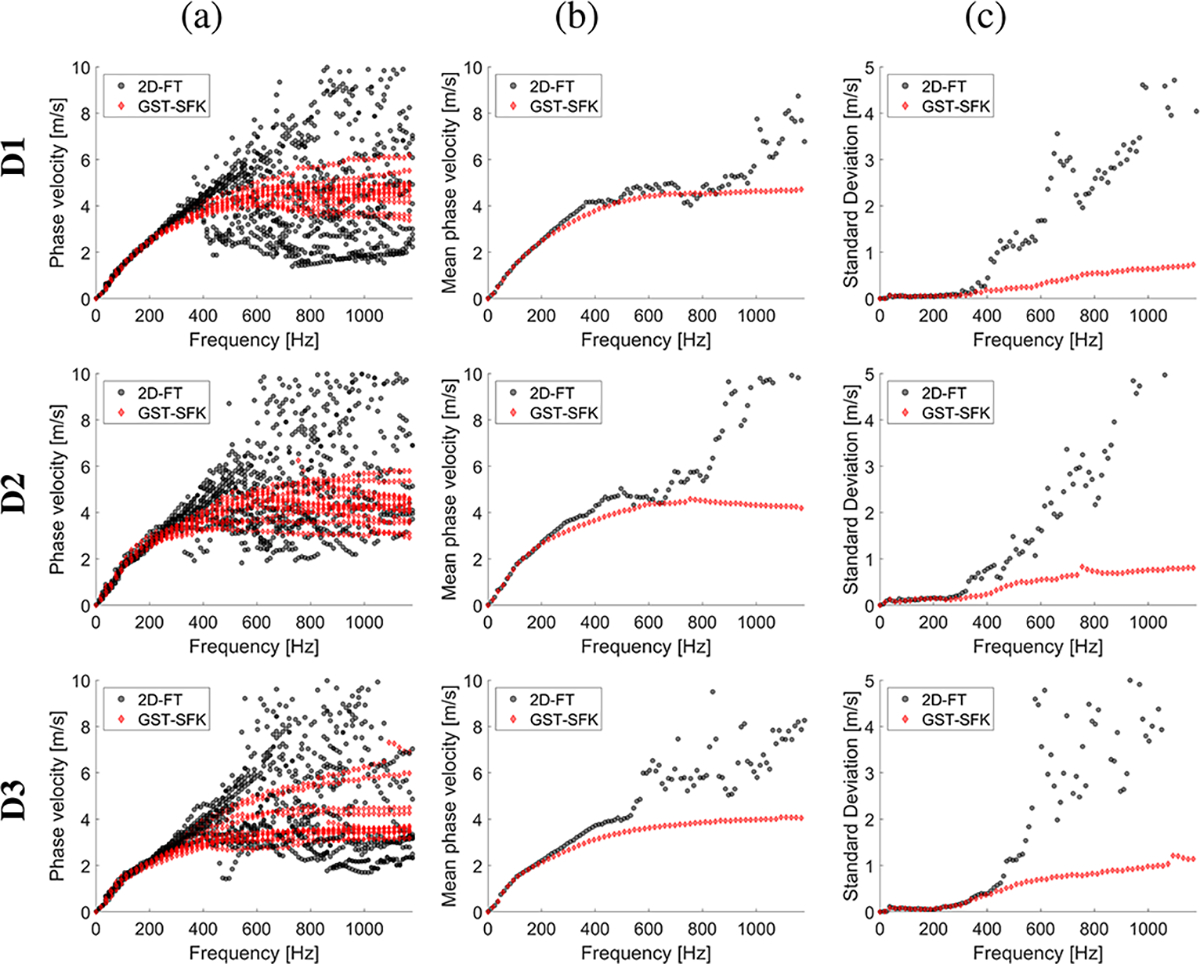
Phase velocity curves calculated for the 2D-FT (black dots), and GST-SFK (red diamonds) methods. Results were computed for the in vivo renal transplants, for subjects with IFTA and inflammation (Group D). Results were calculated for (a) 13 data acquisitions. (b) mean, and (c) standard deviation (SD) were calculated on the basis of all acquisitions from (a).

The experimental in vivo renal transplant data were investigated using the GST-SFK approach and the 2D-FT method for shear wave phase velocity estimation, for clinical applications. Results for these two methods were compared and evaluated. Four groups of subjects were examined as discussed in Section II-D, and summarized in Table I. Spatiotemporal shear wave particle velocity signals for one subject from each group, and the two-dimensional, normalized by wavenumber maxima f-k distribution maps, as well as, two-dimensional phase velocity results, with marked maxima of the phase velocity were shown in Fig. 3. Results for additional subjects were shown in the Supplementary Material in Figs. S7, S11, S15, S19. Phase velocity curves calculated for the 2D-FT and GST-SFK methods, for 10–13 data acquisitions, were evaluated. Results were summarized in Figs. 4, 5, 6, and 7, for groups A-D, respectively. Individual phase velocity dispersion curves for all data acquisitions were plotted in sub-figures (a). The mean and SD, calculated on the basis of all acquisitions, were shown in sub-figures (b) and (c), respectively.

Similar performance between the 2D-FT and GST-SFK methods can be seen for frequencies up to about 300 Hz. Then, the 2D-FT approach estimated dispersion curves were highly scattered within the measured acquisitions, making calculated results above 300 Hz unreliable. The mean phase velocity curves had a discontinuous trend which is unrealistic for physical media. Additionally, the SD curves showed a sharp increase in the SD value (often exceeding 2 m/s and more), e.g., starting at 300 Hz for subject A1, and starting from 400 Hz for subjects A2, and A3, respectively, from the group of healthy subjects, A. Similarly, a sudden increase in SD occurred in other renal transplant subjects and the 2D-FT method, for which similar cut-off frequencies can be evaluated.

On the other hand, the GST-SFK method outperformed the 2D-FT approach in higher frequency ranges. Variation of the GST-SFK dispersion curves, in sub-figures (a), was lower than for the 2D-FT. The SD curves for GST-SFK up to the above-mentioned cut-off frequencies were approximately on the same level as for 2D-FT. Then, with increasing frequency the GST-SFK method outperformed the second technique. The GST-SFK was able to maintain estimated phase velocity curves with SD values below 1 m/s for twice the frequency range and more. For an example, considering the same healthy subjects as above, SD values for subject A1 were below 1 m/s up to 920 Hz, and up to approximately 1200 Hz (the Nyquist frequency) for subjects A2, and A3, respectively. In addition, most examined subjects did not exhibit discontinuous SD curves for GST-SFK, as opposed to the 2D-FT approach.

**Fig. 8.**
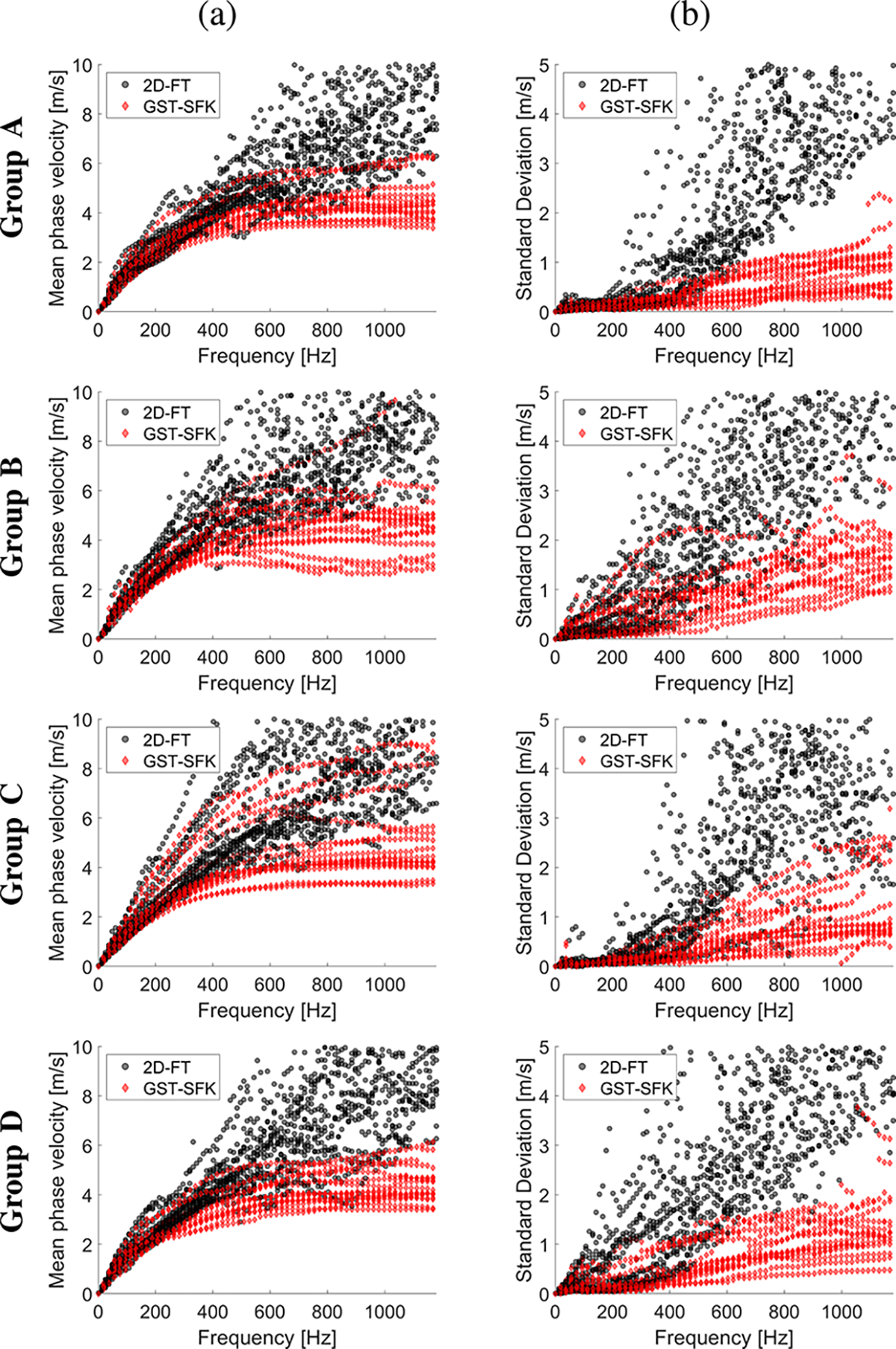
Phase velocity curves calculated for the 2D-FT (black dots), and GST-SFK (red diamonds) methods. Results were computed for the in vivo renal transplants, for subject groups A–D. All groups consisted of 15 subjects each. Results were calculated for (a) mean, and (b) standard deviation (SD). (a) Mean. (b) SD.

**Fig. 9.**
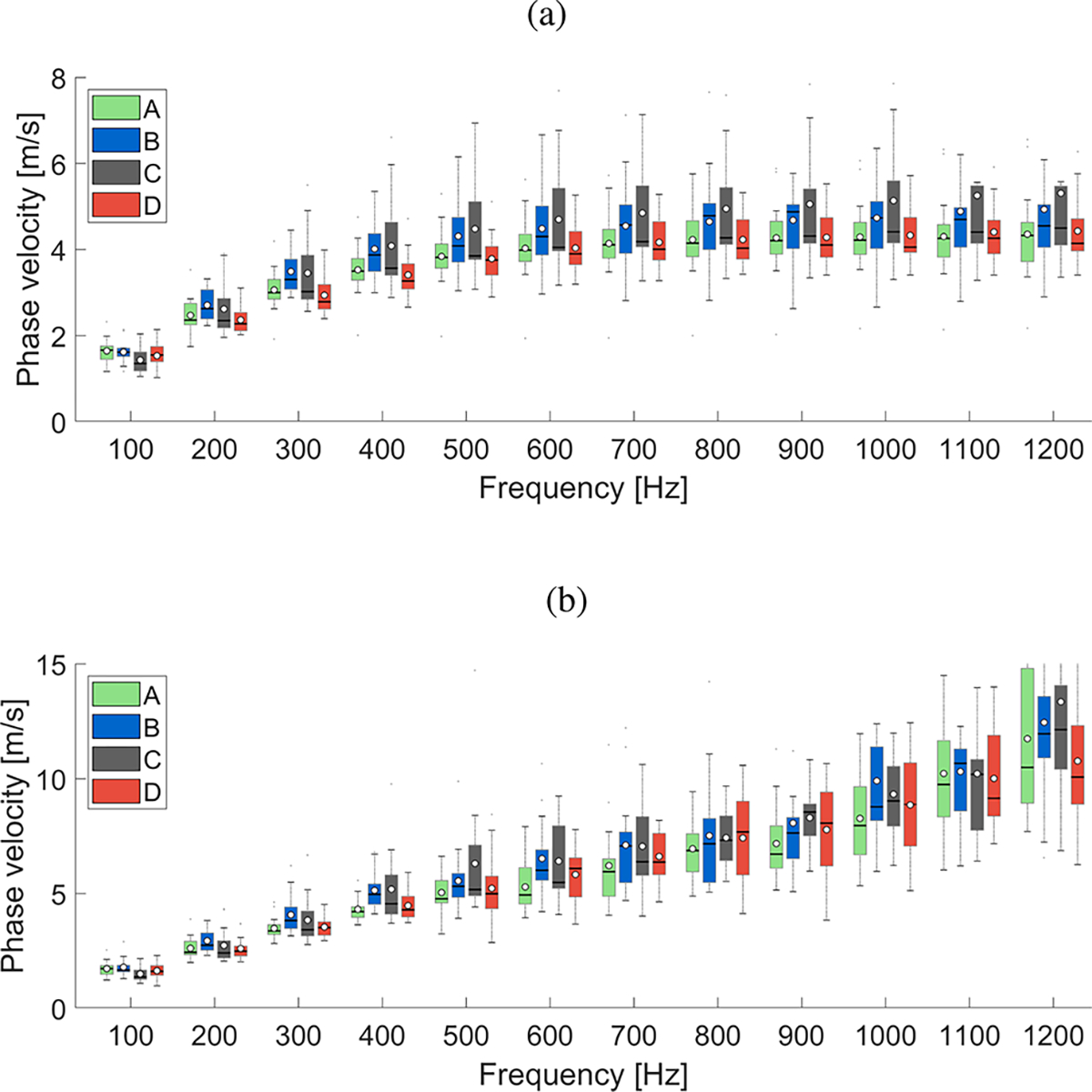
Box plots calculated for the phase velocity computed in a frequency range from 100–1200 Hz. White circles represent mean values, whereas a solid line within the box corresponds to a median value. Phase velocity was computed for the in vivo renal transplant data, for subject groups A-D. All groups consisted of 15 subjects each. Results were presented for the (a) GST-SFK, and (b) 2D-FT methods, respectively. Note the different vertical ranges for each plot.

Fig. 8 shows the mean phase velocity and the SD curves for the four groups described in Table I. Box plots of the phase velocity, for the four subject groups investigated, were calculated for the frequency range from 100–1200 Hz, with an interval of 100 Hz. Results were summarized in Fig. 9 for the GST-SFK method (Fig. 9(a)), and the 2D-FT approach (Fig. 9(b)).

Box plots became more separated within the investigated subjects groups with increasing frequency for the GST-SFK method. Similar separation was not observed for the 2D-FT method in Fig. 9(b). Mean values in the box plots became much wider for frequencies starting from 500 Hz and they were at a similar level between each subject group. It is worth noting that different phase velocity ranges in Fig. 9(a) and (b) were used.

**Fig. 10.**
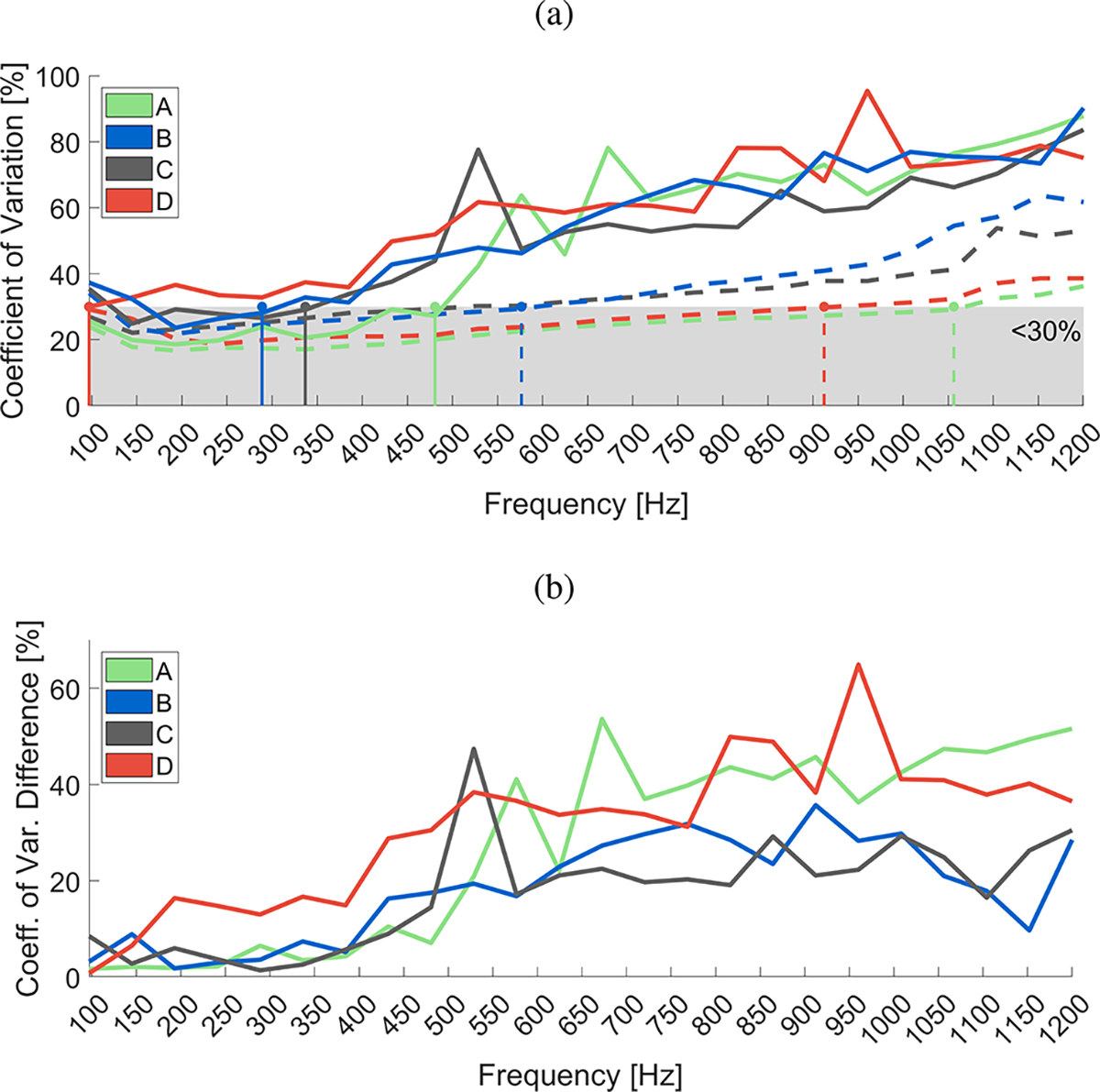
(a) Coefficient of variation (CV) calculated for the phase velocity computed in a frequency range from 100–1200 Hz, for the GST-SFK (dashed lines), and 2D-FT (solid lines) methods, respectively. The shaded gray area corresponds to the CV < 30%, and horizontal lines indicate frequency values before exceeding that region (1050, 550, 450, and 900 Hz, for the GST-SFK approach and subject groups A-D, and 450, 250, 300, and 100 Hz for the same subject groups, and the 2D-FT method). (b) CV difference between the two methods. Phase velocity was computed for the in vivo renal transplant data, for subject groups A-D. All groups consisted of 15 subjects each.

Fig. 10(a) shows the CV calculated for the phase velocity, computed in a frequency range from 100–1200 Hz, for the GST-SFK (dashed lines), and 2D-FT (solid lines) methods, respectively. The shaded gray area corresponds to CV < 30%, and horizontal lines indicate maximum frequency values before exceeding that region. The localized maximum frequency values for CV < 30% were: 1050, 550, 450, and 900 Hz, for the GST-SFK approach and subject groups A-D, whereas for the 2D-FT method these frequencies were 450, 250, 300, and 100 Hz for the same subject groups, respectively. The frequency range meeting this criterion was approximately twice as high for GST-SFK as for 2D-FT, for subject groups A, B, and C. For subject group D, this criterion was not met for 2D-FT.

Coefficient of variation difference between the two methods is shown in Fig. 10(b). The CV for frequencies up to 400 Hz was at approximately the same level, i.e. 25±5% for the two methods investigated and subject groups A, B, and C. At the same time, the CV difference for subject group D and between the two methods was at the level of ~ 15%. Above 400 Hz, higher separation of CV between the GST-SFK and 2D-FT methods was present, with the highest CV difference observed for subject groups A and D. Groups B and C exhibited lower CV difference between the two methods but still, the CV values for the GST-SFK method were almost two times lower than for the 2D-FT approach.

Findings from the statistical analysis suggest that the using a multivariable approach for three-factor ANOVA with two repeated factors is appropriate (*p*-value from Mauchly’s sphericity test < 0.0001). For testing the main effects, there was no statistically significant differences between the groups (*p* = 0.2530). However, the main effects of method (*p* < 0.0001) and frequency(*p* < 0.0001) were statistically significant. This suggests that the phase velocity values were higher for the 2D-FT compared to the GST-SFK, when adjusted for group and frequency and outcome values were statistically significantly different at various frequencies when adjusted for group and method. While testing interaction terms, only interactions between method and frequency were statistically significant suggesting that the outcome values were significantly different among various combinations of method and frequency. Other two-way interactions and a three-way interaction were not statistically significant (all *p* values > 0.05).

Plots of the coefficient of variation suggest that the variability relative to mean is higher for the 2D-FT compared to the GST-SFK method, and the coefficient of variation generally tends to increase at higher frequency values.

**Fig. 11.**
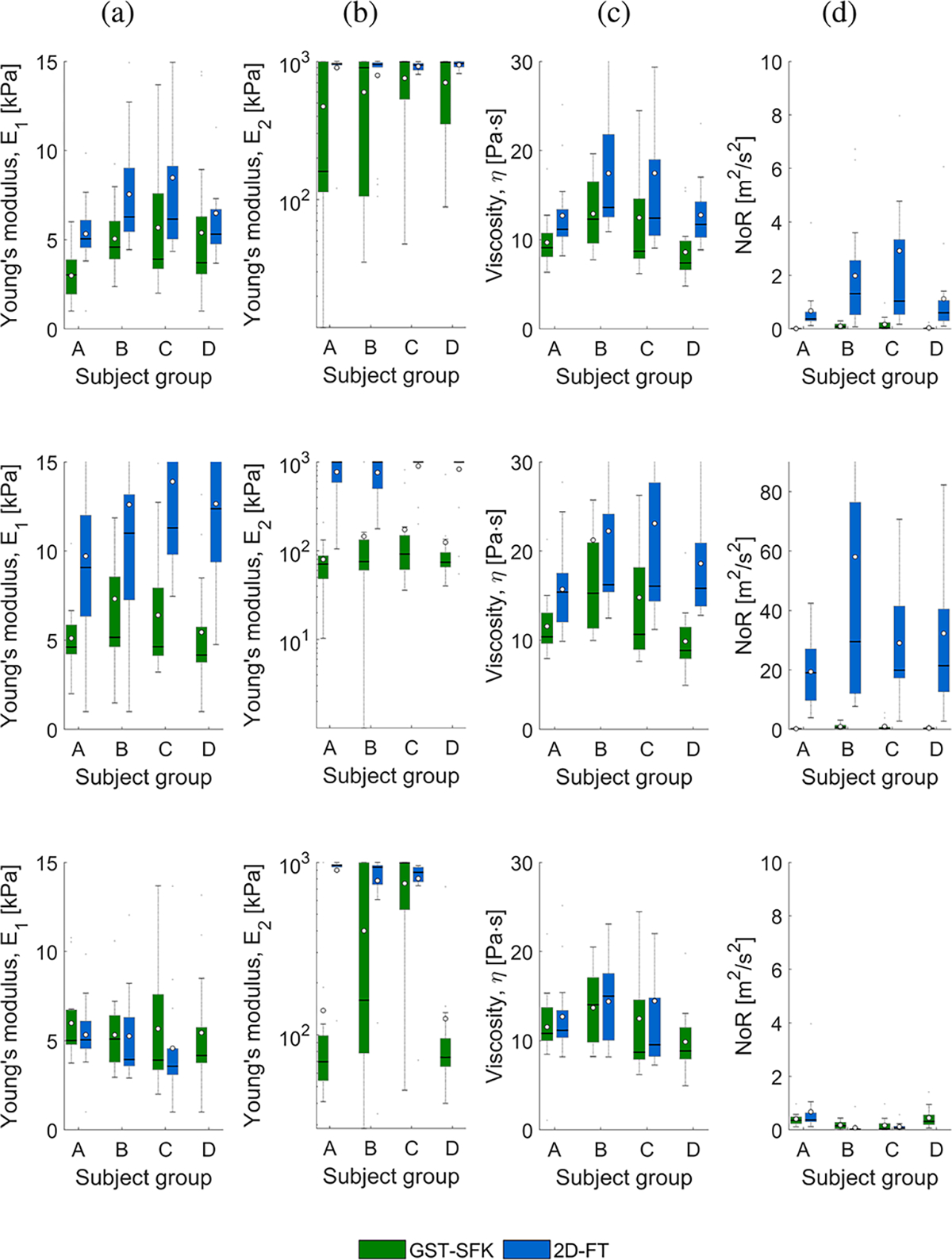
Box plots calculated for estimated Zener parameters (a) Young’s modulus, $E_{1}$, (b) Young’s modulus, $E_{2}$, (c) viscosity, η, and (d) the norm of residuals, NoR, for GST-SFK and 2D-FT methods. White circles represent mean values, whereas a solid line within the box corresponds to a median value. A fixed frequency range of 200–450 Hz was used for the Zener fit in the top row, for both techniques, where all groups (except D for 2D-FT) had CV < 30% (Case 1, top row). The middle row presents the Zener fit for frequency range of 200–900 Hz (Case 2, middle row). The bottom row shows the Zener fit for frequency range starting from 200 Hz up to the maximum frequency for which CV < 30% for a given subject group and given approach (Case 3, bottom row). Results are presented for the in vivo renal transplant data, for subject groups A–D. All groups consisted of 15 subjects each.

Based on the phase velocity curves estimated for all the subject groups and acquisitions, Zener viscoelastic parameters were estimated, using frequency ranges used in Cases 1–3, and the results are presented in Fig. 11. Similar as for the TM viscoelastic phantoms, box plots were calculated for (a) Young’s modulus, $E_{1}$, (b) Young’s modulus, $E_{2}$, (c) viscosity, η, and (d) the norm of residuals, NoR, for GST-SFK and 2D-FT methods, and the results were grouped by subject type. The lower the NoR value, the better the accuracy of the fit of the rheological Zener model to the data. This in turn, gives the more reliable $E_{1},\;E_{2}$, and η estimates in Fig. 11(a), (b), and (c).

The *p*-values resulting from the Wilcoxon rank sum test, which was performed on the estimated Zener parameters are presented in Table II. For Case 1, three statistically significant groups were identified for the GST-SFK approach, while the p-value was below 0.05 for comparing groups A-B and $E_{1}$, and η for the 2D-FT approach. For Case 2, both methods had *p* < 0.05 for groups A-B for η. The GST-SFK method yielded *p*-values below 0.05 for group A-C in Case 3 for Young’s modulus $E_{2}$. In contrast, for the 2D-FT method two groups were observed with statistically significant estimates, i.e., A-C for $E_{1}$ and η. None of the tested methods showed statistically significant results comparing groups A vs D.

## IV. Discussion

In this research work, we investigated a recently proposed approach, called GST-SFK, for robust calculation of shear wave phase velocity in viscoelastic phantoms and in vivo renal transplants. The GST-SFK method was assessed with shear wave particle velocity data induced by ARF. Data were acquired for 15 and 10–13 spatial locations, for each examined phantom and subject, respectively. In the previous work, the GST-SFK method was initially tested on single acquisitions for TM phantoms [35]. Custom-made TM phantoms were tested on a research ultrasound platform resulting in an effective frame rate of 4.167 kHz, whereas the in vivo renal transplants were studied using a clinical scanner with almost two times lower sampling rate, i.e., 2.412 kHz. The robustness of the GST-SFK method was tested based on multiple acquisitions and calculations of the mean phase velocity and standard deviation.

The results of the mean phase velocity for the TM phantoms were similar for both the GST-SFK and 2D-FT methods, for the first part of the frequency range, i.e. up to 400 Hz for Phantom I, 1450 Hz for Phantoms II and III, 1300 Hz for Phantom IV, and 700 Hz for Phantom V. After these frequency ranges, only the GST-SFK approach gave stable results with very low SD values below 0.10 m/s, making the entire frequency band useful and trustworthy. The observed SD was caused by the fact that all fifteen data acquisitions for each phantom were acquired at different spatial positions, and the upper phantom scanning area was 7.5 × 18 mm for rectangular Phantom III, and 11 cm diameter for other cylindrical-shaped phantoms. All acquisitions were acquired in random locations that covered the entire phantom scanning area. The true level of dispersion or level of viscoelasticity was unknown but expected to insignificantly vary for custom-made phantoms [36].

The results for the in vivo renal transplants showed greater variation compared to the TM viscoelastic phantoms for the following reasons. First, the examinations were carried out by experienced clinical sonographers, and subjects were asked to hold still during the examination but this did not prevent all movement of the kidney. Moreover, all 10–13 acquisitions were made in a loop after having previously located the ARF beam in the renal cortex in the middle of the kidney in a longitudinal plane. Every movement of the body caused the ARF beam to move from the previously set position. Therefore, each acquisition could potentially contain varying amounts of information derived from the renal cortex and other tissue parts, such as the renal fascia or medulla. Hence, the clear differences for the estimated dispersion curves between consecutive data acquisitions of the same subjects. Second, the spatiotemporal shear wave motion data measured for renal transplants had a shorter lateral segment length compared to the TM phantoms, and the curvilinear probe was used with a resulting spatial resolution of 0.2396 mm, while for the linear probe and TM phantoms it was 0.154 mm. Shorter data records may generally produce higher variation [19]. Third, the spatial window for the shear wave motion data selection was manually chosen during the processing to make sure the data was in the cortex. This could have caused additional discrepancies in the final results. Fourth, almost two times lower sampling rate was used to record the data, again reducing the quality of the recorded shear wave motion data. Fifth, the directional filtering used to separate leftward from rightward traveling shear waves could produce some artifacts. Sixth, the lateral segment length was shorter for the in vivo case compared to the CIRS phantoms. Seventh, the study population was limited to mild to moderate categories of disease.

With all of the above factors in mind, noticeable standard deviation of the phase velocity was expected for examined subjects. Nevertheless, dispersion curves estimated using the GST-SFK approach significantly improved the results compared to the 2D-FT technique, reducing the standard deviation several times for frequencies close to 800 Hz and above. The level of the observed standard deviation most likely comes from the movement of the tissue and the additional factors described in the previous paragraph.

In addition, in this work, box plots were plotted and the coefficient of variation was examined on 60 subjects after renal transplant, based on the GST-SFK and 2D-FT phase velocity results. The obtained results confirmed better robustness (lower SD and NoR values) of the GST-SFK method over the 2D-FT method, providing more stable results, which has a great promise in clinical applications.

To ensure that the Zener model is an appropriate rheological model for the data sets investigated in this work, we conducted a convergence analysis in the frequency domain. The purpose of this analysis was to assess the stability of the Zener model curve fit and examine the behavior of its parameters, namely $E_{1},\;E_{2}$, and η. We initiated the convergence analysis by fitting the Zener model to the data within an initial frequency range of 200–300 Hz. Subsequently, we incrementally extended the frequency range by 25 Hz up to 2000 Hz for the CIRS phantoms and up to 1100 Hz for the in vivo renal transplants. A convergent model should exhibit parameters that stabilize at a constant level and remain unchanged as the frequency range increases, with the aim of achieving the lowest NoR values. Therefore, we conducted a thorough examination of the behavior of $E_{1},\;E_{2}$, and η as the frequency range expanded. The analysis focused on observing if these parameters reached a plateau and maintained consistent values, indicating convergence of the Zener model to the data sets under investigation. Additionally, we evaluated the NoR values associated with each parameter estimation to quantify the goodness of fit and ensure that they were minimized as the frequency range increased. If the NoR values were found to be higher than 1 m^2^/s^2^, indicating that the curve fit was not sufficiently accurate, we considered the estimated Zener model parameters to be unreliable. In such cases, where the NoR values exceeded the threshold, we did not consider the obtained parameter values to be trustworthy. This approach ensured that only robust and accurate parameter estimates were taken into account for further analysis and interpretation. Similar investigation was performed for two parameter the Kelvin-Voigt (KV) model, and the results were summarized in the Supplementary Material.

**Fig. 12.**
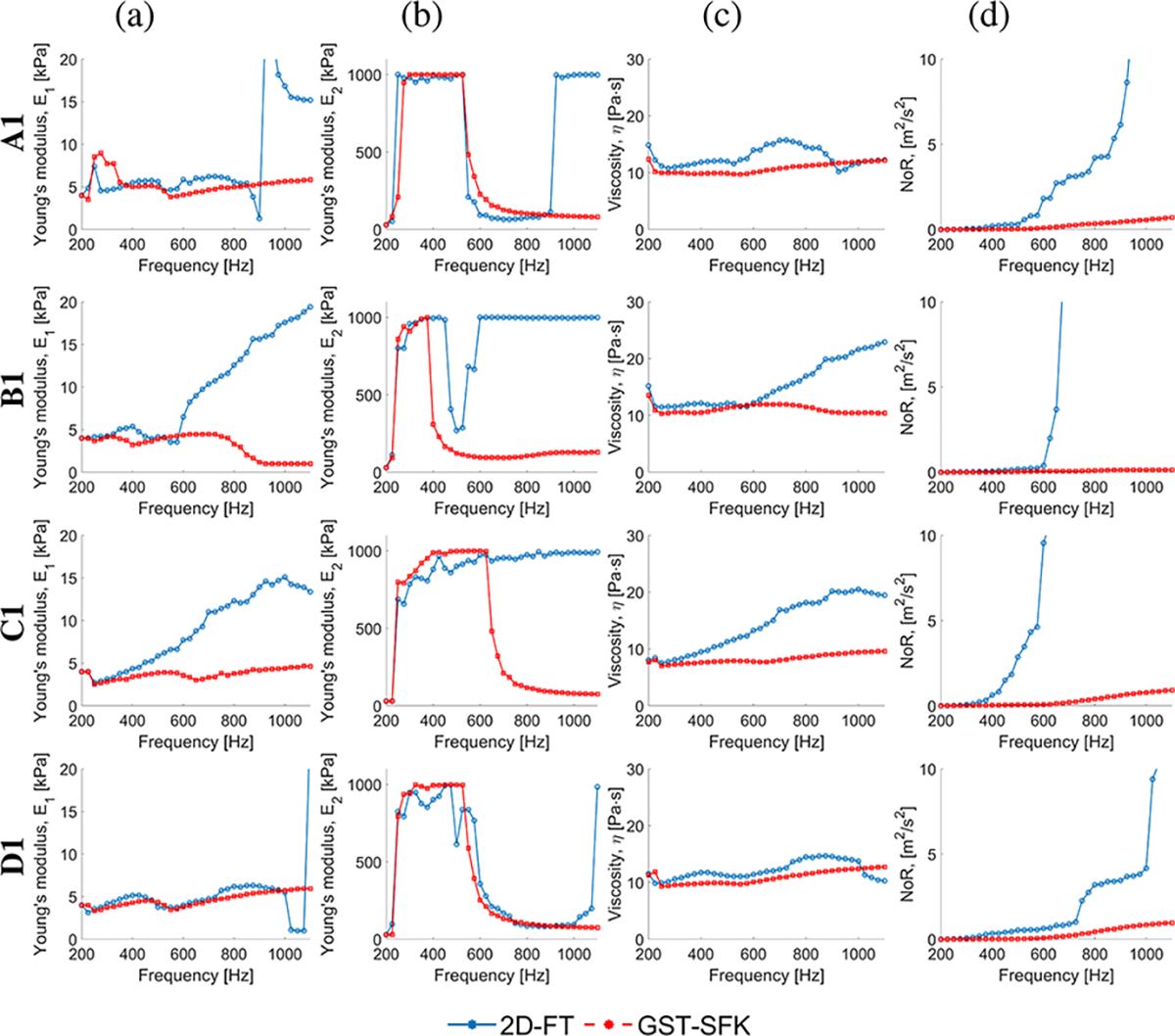
Convergence analysis of the Zener model fit. Results were computed for the in vivo renal transplants, for sample data acquisitions. The mean phase velocity curves obtained using the 2D-FT and GST-SFK methods were used for fitting. (a) $E_{1}$. (b) $E_{2}$. (c) η. (d) NoR.

Fig. 12 illustrates sample convergence plots of the Zener model fit for selected in vivo renal transplants, while additional plots can be found in the Supplementary Material. The convergence analysis was conducted based on the mean phase velocity curves obtained using the 2D-FT and GST-SFK approaches.

**Fig. 13.**
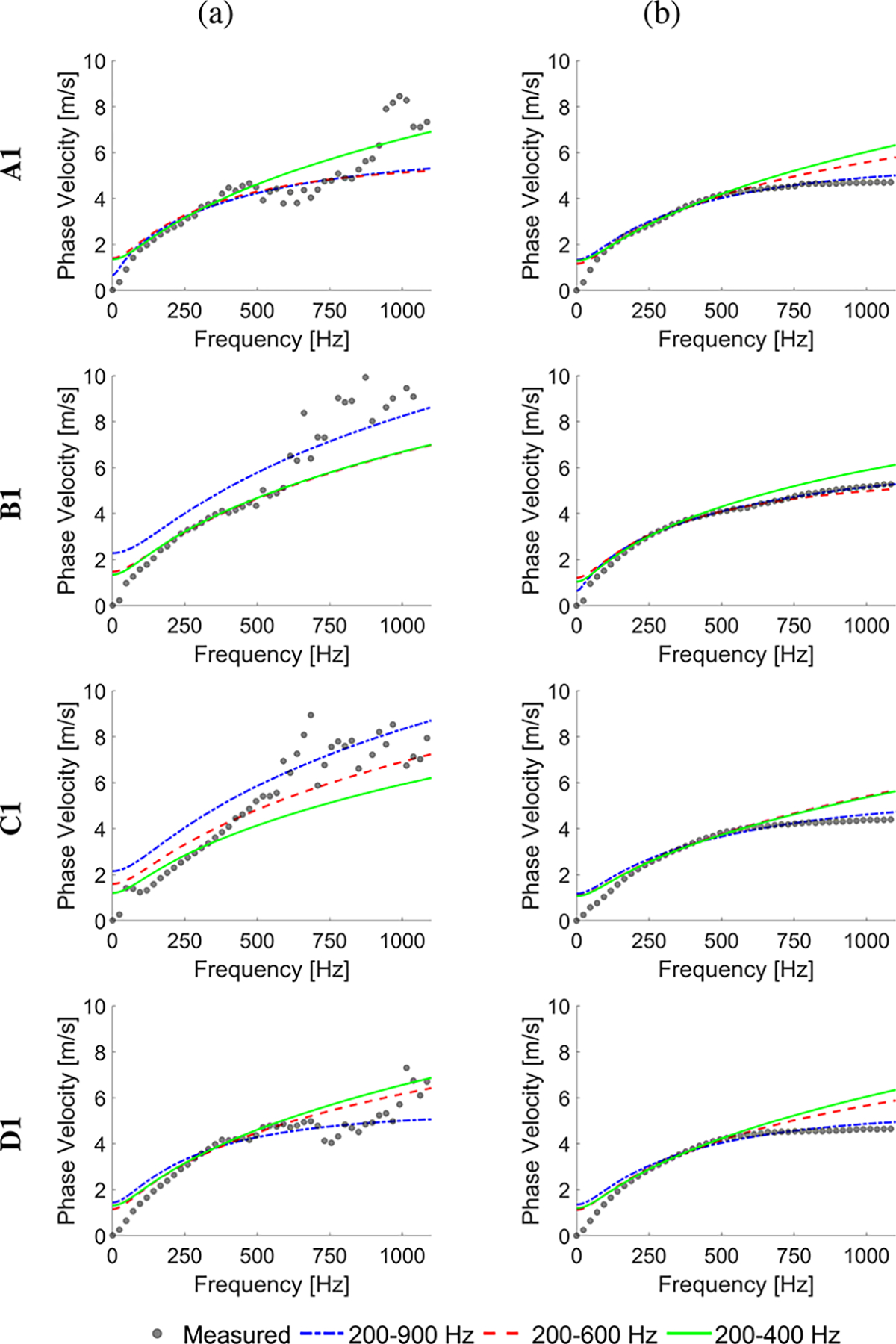
Mean phase velocity dispersion curves (gray dots) measured using the (a) 2D-FT and (b) GST-SFK methods. Each figure contains fitted analytical phase velocity curves calculated using the Zener model for various frequency ranges, i.e.: 200–400 Hz, 200–600 Hz, and 200–900 Hz. Results were computed for the in vivo renal transplants for subjects.

The analysis revealed that the $E_{2}$ parameter exhibited the highest variation across the entire frequency range tested compared to other parameters. Specifically, for shorter frequency ranges (e.g., < 600 Hz for A1, < 400 Hz for B1, etc.), the $E_{2}$ parameter exhibited significantly higher values, indicating that the behavior of the Zener model resembled that of the KV model. In such cases, the KV model appeared to be also appropriate, as evidenced by the very low NoR values observed within these frequency ranges. This observation was supported by the convergence analysis performed for the KV model, which is presented in the Supplementary Material. However, when considering a wider frequency range, the KV model is no longer relevant, and the Zener model becomes more appropriate. In this wider range, all three parameters of the Zener model begin to stabilize having low NoR values, indicating convergence. Therefore, it is important to consider the frequency range under investigation to determine the appropriate rheological model, with the Zener model demonstrating suitability for wider frequency ranges compared to the KV model. Sample fitted analytical phase velocity curves calculated using the Zener model for various frequency ranges are shown in Fig. 13. Readers are referred to the Supplementary Material where additional results were presented.

Based on the phase velocity curves estimated using the 2D-FT method, one could possibly select a frequency range from 200 up to 400 Hz (Figs. 4, 5, 6, and 7). For this frequency range, the *E*_1_ values for healthy subjects (A) were better separated from other groups for the GST-SFK approach compared to the 2D-FT method (Fig. 11, top row). In addition, the NoR parameter for the 2D-FT approach was higher than for GST-SFK meaning that the estimates for the 2D-FT method should be trusted less. The $E_{2}$ values had larger boxplots for the GST-SFK approach compared to the 2D-FT method. However, if we broaden the frequency range for the Zener fitting (Case 2, where the Zener fit was performed for frequency range of 200–900 Hz), we note that the NoR parameter was higher than 20 m^2^/s^2^ for the 2D-FT approach and we can not trust the estimated viscoelastic parameters for this method. In contrary, the GST-SFK exhibited NoR below 1 m^2^/s^2^ making the estimates more reliable. If we make the frequency range dependent on the CV coefficient (Case 3: from 200 Hz up to the maximum frequency for which CV < 30% for a given subject group and given approach), the separation of $E_{2}$ values between groups A-C was statistically significant for the GST-SFK method, and both Young’s moduli were statistically significantly different for the 2D-FT approach. The η parameters were not statistically significantly different (*p* < 0.05) for any of the method, for the extended frequency range used. Noteworthy, the phase velocity curves for the subject group D estimated using the 2D-FT approach did not have CV < 30%, hence it could not be used for KV fitting (Case 3, bottom row in Fig. 11). In addition, the goodness of Zener fit was much better for the GST-SFK approach, for limited frequency range tested. The NoR values were similar across the groups of different subjects for the GST-SFK.

Changes to $E_{1},\;E_{2}$ and η have been noticed along with the frequency range, which may raise the question of which set of values and frequency range is trustworthy. Both estimates are correct, for the shorter and larger frequency ranges, if we keep in mind what bandwidth was used for the Zener fitting. The shorter frequency range has less information about frequency-dependent phase velocity. The $E_{1},\;E_{2}$ and η estimates for shorter and larger frequency ranges may differ as different amounts of information were taken into account for Zener fitting. From these observations, we can conclude that the phase velocity at higher frequencies contains information that allows better separation of $E_{1},\;E_{2}$ and η between groups.

The main difference between the $E_{1},\;E_{2}$ and η estimates for CIRS and the kidney data is that for the TM phantoms they were estimated from different acquisitions, but for the kidney data $E_{1},\;E_{2}$ and η were estimated from different patients.

In this work, we tested the robustness of our method based on four groups of subjects including healthy subjects and subjects with present IFTA and/or inflammation. This shows that the GST-SFK approach can be further applied to SWE data from different stages of kidney health for its noninvasive diagnosis, which is out of the scope of this work, due to the limited amount of data.

For each experimental data set, extended usable bandwidth with lower standard deviation and the CV, was observed for GST-SFK. Having shear wave phase velocity responses for wider frequency band improves rheological models responses compared to the limited frequency ranges, as noticed for instance in [15], [37]. This translates into a statistically significant separation of Zener parameters and lower norm of residuals achieved for the Zener rheological model, for the GST-SFK approach compared to the 2D-FT method, as shown in this work. Therefore, the GST-SFK approach is a very promising tool which can be combined with various rheological models for better in vivo renal transplants characterization and to discriminate healthy versus diseased tissue, which is also out of the scope of this work. This could be extended for use in other soft tissues and for characterizing shear wave dispersion slope over a larger bandwidth [38], [39], [40]. In addition, robustness of the GST-SFK method could be adopted for Local Phase Velocity Imaging (LPVI) method in order to improve two-dimensional mapping for higher frequencies [14], [41], [42].

The vast majority of available research has focused on characterizing the viscoelastic properties of the in vivo kidney using the KV model fitting approach [43], [44], [45], [46], [47], [48], [49]. The Aixplorer Mach 30 ultrasound scanner from Hologic was utilized to estimate shear elasticity and viscosity within the ranges of 5–10 kPa and 2–3 Pa · s, respectively [43], [44], [45]. Some limited data also exist, employing the shear dispersion ultrasound vibrometry (SDUV) method, which involves generating shear waves at frequencies that are multiples of 100 Hz [46], [47], [48], [49]. The reported range of viscoelastic parameters obtained through SDUV aligns closely with the values reported by Maralescu et al. in human studies [43], [44], [45].

In our work, we adopted a different approach based on convergence analysis and employed the Zener viscoelastic model to deduce viscoelastic parameters. It is important to note that the Zener model has distinct interpretations compared to the KV model. However, when comparing the estimated dispersion phase velocity curves in our study to those reported in [46], [47], we observe similar results, particularly when examining the same frequency ranges. This suggests that, our findings are consistent with existing literature, further contributing to our understanding of the viscoelastic properties of the in vivo kidney within the context of the frequency ranges examined.

The computational time for the in vivo subjects, after shear wave motion reconstruction, was approximately 4.3 seconds for dispersion curves calculation using GST-SFK and a MATLAB R2020a (Mathworks, Natick, MA) implementation on a standalone PC. This computational time may need reduction before implementing the GST-SFK method on a clinical ultrasound scanner, which is one of the current limitations of this method. Sixty subjects were investigated in this work, and the groups were relatively small, which is one of the limitations of this work.

## V. Conclusion

This work demonstrates the S-transform-based (GST-SFK) method robustness for the evaluation of shear wave phase velocity dispersion curves used in clinical applications. The method was examined on experimental viscoelastic phantom data as well as on in vivo renal transplant tissue data. Fifteen data acquisitions per phantom and 10–13 data acquisitions per in vivo data were used to test the performance of the method. Compared to the 2D-FT, the GST-SFK approach obtained better performance and extended usable bandwidth for all the data examined. More importantly, phase velocity variance was highly reduced and SD curves did not exhibit discontinuities over frequency range. It is noteworthy that the fitting of the rheological model should be preceded by a convergence analysis in order to be able to estimate which model best represents the measured dispersion curves and in which frequency range. The GST-SFK method could be used in further in vivo renal transplant data evaluation for assessment of chronic allograft rejection, and other soft tissues characterization.

## Supplementary Material

supp1-3323983

## Figures and Tables

**TABLE I:** In Vivo Renal Transplant Data Divided Into Four Groups Based on the Inflammation and Interstitial Fibrosis and Tubular Atrophy (IFTA) Presence

Subject Group	IFTA	Inflammation	Group velocity, MEAN±SD [m/s]

A	No	No	2.35 ± 0.49
B	No	Yes	2.53 ± 0.70
C	Yes	No	2.39 ± 0.81
D	Yes	Yes	2.26 ± 0.38

Group a corresponds to healthy subjects. All groups consisted of 15 subjects each.

**TABLE II:** *p*-Values Resulting from the Wilcoxon Rank Sum Test, Which Was Performed on the Estimated Zener Parameters Presented in Fig. 11

Phantom	Method	Young’s modulus, *E*_1_			Young’s modulus, *E*_2_			Viscosity, *η*		

		A-B	A-C	A-D	A-B	A-C	A-D	A-B	A-C A-D

**Case 1**	**GST-SFK**	0.003	0.017	0.065	0.663	0.141	0.178	0.024	0.724	0.178
	**2D-FT**	0.044	0.101	0.494	0.756	0.310	0.663	0.015	0.254	0.820

**Case 2**	**GST-SFK**	0.237	0.633	0.520	0.548	0.254	0.520	0.008	0.633	0.093
	**2D-FT**	0.633	0.078	0.120	0.852	0.663	0.917	0.044	0.141	0.178

**Case 3**	**GST-SFK**	0.443	0.110	0.120	0.071	0.001	0.419	0.290	0.290	0.093
	**2D-FT**	0.330	0.033	—	0.165	0.002	—	0.419	0.290	—

Case 1: Upper Limit: 400 Hz;

Case 2: Upper Limit: 900 Hz;

Case 3: Upper Limit: CV < 30%.

Results are reported for the in *vivo* renal transplant data. Shaded areas highlight *p*-values below 0.05.
