# Supplementary material for: Evaluation of Robustness of S-Transform Based Phase Velocity Estimation in Viscoelastic Phantoms and Renal Transplants
Source: IEEE Trans Biomed Eng. Author manuscript; Available in PMC 2024 Mar 18. (PMC10947612; doi:10.1109/TBME.2023.3323983)
Supplement: supp1-3323983 [file NIHMS1970319-supplement-supp1-3323983.pdf]

Evaluation of Robustness of S-transform based Phase Velocity Estimation in Viscoelastic Phantoms and Renal Transplants - Supplementary Material

Piotr Kijanka, *Member, IEEE*, Luiz Vasconcelos, *Member, IEEE*, Jay Mandrekar, and Matthew W. Urban, *Senior Member, IEEE*

THIS document serves as the Supplementary Material for the main manuscript titled *Evaluation of Robustness of S-transform based Phase Velocity Estimation in Viscoelastic Phantoms and Renal Transplants* by Piotr Kijanka, Luiz Vasconcelos, Jay Mandrekar and Matthew W. Urban submitted to IEEE Transactions on Biomedical Engineering.

I. Viscoelastic Rheological Models

Based on the phase velocity estimates over a given frequency range, the elasticity and viscosity parameters were deduced. In this work, we used the Kelvin-Voigt (KV) rheological viscoelastic model, as a comparison to the Zener model introduced in the main manuscript. The KV model is composed of a dashpot and a spring arranged in parallel. The stress-strain relationship of the KV model is given in the form

$$\sigma = \left(\mu_1 - \mu_2 \frac{\partial}{\partial t}\right)\varepsilon, \tag{S1}$$

where the stress, σ, is related to the strain, ε, by the shear elasticity μ_1, the shear viscosity μ_2, and the time derivative operator ($\partial/\partial t$). After analyzing the equation of wave motion, and solving the complex wave vector for the 1-D Helmholtz equation, the shear wave velocity for the KV model can be calculated as

$$V_s(\omega) = \sqrt{\frac{2\left(\mu_1^2 + \omega^2\mu_2^2\right)}{\rho\left(\mu_1 + \sqrt{\mu_1^2 + \omega^2\mu_2^2}\right)}} \tag{S2}$$

where ρ stands for the density and ω is an angular frequency, i.e., $\omega = 2\pi f$.

In order to estimate μ_1 and μ_2 parameters, $V_s(\omega)$ was estimated using a nonlinear least-squares problem (NLSQ) in a form

$$[\mu_1, \mu_2] = \min_{\mu_1,\mu_2} ||V_s(f) - V_{ph}(f)||_2^2. \tag{S3}$$

Equations (S3) and (8), in the main manuscript, were numerically solved using the MATLAB solver *lsqcurvefit*. The KV and Zener fits were done using two sets of frequency ranges: a short one (with reduced variability and SD) that one would select based on the phase velocity curves estimated using the 2D-FT method, and an extended frequency range. The short frequency range used for the KV fit and the TM phantoms was: 150-400 Hz for Phantom I, 150-1400 Hz for Phantom II, 150-1400 Hz for Phantom III, 150-700 Hz for Phantom IV, and 150-600 Hz for Phantom V. The extended frequency range of 150-1800 Hz for the TM phantoms was used.

Three frequency ranges were used for renal transplants, i.e.:

- Case 1: a fixed frequency range of 200-450 Hz, where all groups (except D for 2D-FT) had a coefficient of variation (CV) $< 30\%$;
- Case 2: a fixed frequency range of 200-900 Hz;
- Case 3: frequency range starting from 200 Hz up to the maximum frequency for which CV $< 30\%$ for a given subject group and given approach.

The coefficient of variation (CV) was defined as $CV = \frac{SD}{MEAN} \cdot 100\%$ and shows the degree of variation relative to the sample mean.

II. TM Phantoms

Shear wave particle velocity motion data for custom-made TM viscoelastic phantoms were examined. Fig. S1a shows shear wave spatiotemporal data measured for five different

This work was supported in part by the National Science Centre in Poland under research project no. UMO-2021/43/D/ST8/01295 and in part by grant R01DK092255, from the National Institutes of Health. The content is solely the responsibility of authors and does not necessarily represent the official views of the National Institute of Diabetes and Digestive and Kidney Diseases or the National Institutes of Health.
(Corresponding author: Piotr Kijanka.)

P. Kijanka is with the Department of Robotics and Mechatronics, AGH University of Krakow, 30-059 Krakow, Poland (e-mail: piotr.kijanka@agh.edu.pl).

L. Vasconcelos is with the Department of Radiology, Mayo Clinic, Rochester, MN 55905 USA.

J. Mandrekar is with the Department of Quantitative Health Sciences, Division of Clinical Trials and Biostatistics, Mayo Clinic, Rochester, MN 55905 USA.

M. W. Urban is with the Department of Radiology, Mayo Clinic, Rochester, MN 55905 USA and also with the Department of Physiology and Biomedical Engineering, Mayo Clinic, Rochester, MN 55905 USA.

Color versions of one or more of the figures in this paper are available online at http://ieeexplore.ieee.org.

TM phantoms. The results for a single acquisition, randomly selected, were displayed, as an example. From Fig. S1a it can be seen that the shear wave velocity is the lowest for Phantom I, and the highest for Phantom V.

Figures S1b and S1c present the frequency-wavenumber (f-k) distribution, also known as k-space which shows the distribution of shear wave energy, reconstructed based on the 2D-FT (Fig. S1b), and GST-SFK (Fig. S1c) methods. The f-k maps were normalized by wavenumber maxima in the frequency direction to highlight the differences between the two methods. Using the f-k maps, phase velocity reconstructions were estimated in Figs. S1d and S1e. The phase velocity maps have superimposed markers corresponding to the maximum peaks of the phase velocity. These results were calculated for the experimental, custom-made TM viscoelastic phantoms I-V, for a randomly selected data acquisitions, which correspond to the shear wave particle velocity motion data shown in Fig. S1a.

Considerable differences can be observed between the results for the 2D-FT method, and the GST-SFK approach. The f-k main energy distributions presented in Figs. S1b and S1c, which determine the shear wave propagation mode in the material, starts diffuse for higher frequencies, i.e. above 500 Hz for Phantom I, and above 1000 Hz for Phantoms II-V. This effect was also seen in the phase velocity reconstructions in Figs. S1d and S1e, where increased magnitude decay for 2D-FT is observed, for above frequencies.

Figures S2 and S4 present the mean phase velocity dispersion curves (depicted as gray dots) obtained from measurements using the 2D-FT (top row) and GST-SFK (bottom row) approaches. Fitted analytical phase velocity curves calculated using the Zener and Kelvin-Voigt (KV) viscoelastic models are overlaid on the data, considering three different frequency ranges. The convergence analysis of the Zener and KV models can be observed in Figs. S3 and S5, respectively.

Comparing the fits of the Zener and KV models, it is evident that the KV model provides reliable fitting for shorter frequency ranges in comparison to the Zener model. This observation is supported by the convergence analysis (low NoR values correspond to good curve fit), which highlights the stability of the KV model parameters in these frequency ranges. The KV model's suitability for shorter frequency ranges indicates that it accurately captures the rheological behavior of the data within that limited frequency range. Furthermore, it is worth noting that the mean phase velocity curves obtained using the 2D-FT method have a reduced usable bandwidth for the rheological model fit compared to the GST-SFK approach. This limitation suggests that the available frequency range for fitting the rheological models is narrower when employing the 2D-FT method. The convergence analysis supports the finding that the KV model is reliable for shorter frequency ranges, while the Zener model provides a more appropriate representation for a wider frequency range.

Figure S6 shows box plots calculated for estimated Kelvin-Voigt parameters, for the GST-SFK and 2D-FT methods. The short and long frequency ranges were used for calculations.

TABLE S1: *In vivo* renal transplant data divided into four groups based on the inflammation and Interstitial Fibrosis and Tubular Atrophy (IFTA) presence. Group A corresponds to healthy subjects. All groups consisted of 15 subjects each.

Subject Group	IFTA	Inflammation	Group velocity, MEAN±SD [m/s]
A	No	No	2.35 ± 0.49
B	No	Yes	2.53 ± 0.70
C	Yes	No	2.39 ± 0.81
D	Yes	Yes	2.26 ± 0.38

III. In vivo Renal Transplants

The experimental *in vivo* renal transplant data were investigated using the GST-SFK approach and the 2D-FT method for shear wave phase velocity estimation, for clinical applications. Results for these two methods were compared and evaluated. Four groups of subjects were examined as discussed in the main manuscript, and summarized in Table S1. Spatiotemporal shear wave particle velocity signals for three subjects from each group were presented in Figs. S7a, S11a, S15a, and S19a, for healthy subjects, subjects with inflammation and no IFTA, subjects with IFTA, but no inflammation, and subjects with IFTA and inflammation, respectively.

The two-dimensional, normalized by wavenumber maxima f-k distribution maps, as well as, two-dimensional phase velocity results, with marked maxima of the phase velocity, were shown in Figs. S7b-S7e, S11b-S11e, S15b-S15e, and S19b-S19e, for all four groups of subjects, A-D, respectively. The differences between the two methods can be distinguished. The f-k distribution maps for the 2D-FT method have disturbance for the main shear wave particle velocity signal which increases with frequency. This, of course, translates into phase velocity maps which are unreliable at higher frequencies, starting at approximately 400 Hz, depending on the subject under consideration. In turn, the GST-SFK method gives much better robustness for corresponding subjects. The f-k distribution maps display the shear wave energy distribution in a stable manner over at least twice the frequency range compared to the 2D-FT. The phase velocity maps from GST-SFK are more homogeneous and have a higher magnitude as a function of frequency compared to 2D-FT, from which the phase velocity dispersion curves for the main shear wave mode can be extracted.

Similar as for the TM viscoelastic phantoms, examples of the fitted analytical phase velocity curves calculated using the Zener and KV models are shown in Figs. S8, S12, S16, and S20. Results for the convergence analysis for the Zener viscoelastic model were shown in Figs. S9, S13, S17, and S21 for subjects A-D, respectively. Alike convergence results, however obtained for the KV model, were summarized in Figs. S10, S14, S18, and S22.

The analysis of the data revealed that the E_2 parameter exhibited the highest variation across the entire frequency range that was tested. Specifically, for shorter frequency ranges (e.g., <600 Hz for A1, <400 Hz for B1, etc.), the E_2 parameter showed significantly elevated values, suggesting a similarity between the behavior of the Zener model and the KV model. In these cases, the KV model also appeared to be suitable, as evidenced by the very low NoR (<0.5 m^2/s^2)

values observed within these frequency ranges. This finding is supported by the convergence analysis conducted specifically for the KV model.

However, when considering a wider frequency range for the GST-SFK approach, the KV model is no longer applicable, and the Zener model becomes more appropriate. Over this broader range, all three parameters of the Zener model demonstrate stabilization (indicating convergence) and exhibit low NoR values (often <1 m^2/s^2). This suggests that the Zener model better captures the rheological properties of the data in this extended frequency range.

Figure S23 shows box plots calculated for estimated KV parameters, which can be compared with the Zener model in Fig. 10, in the main manuscript.

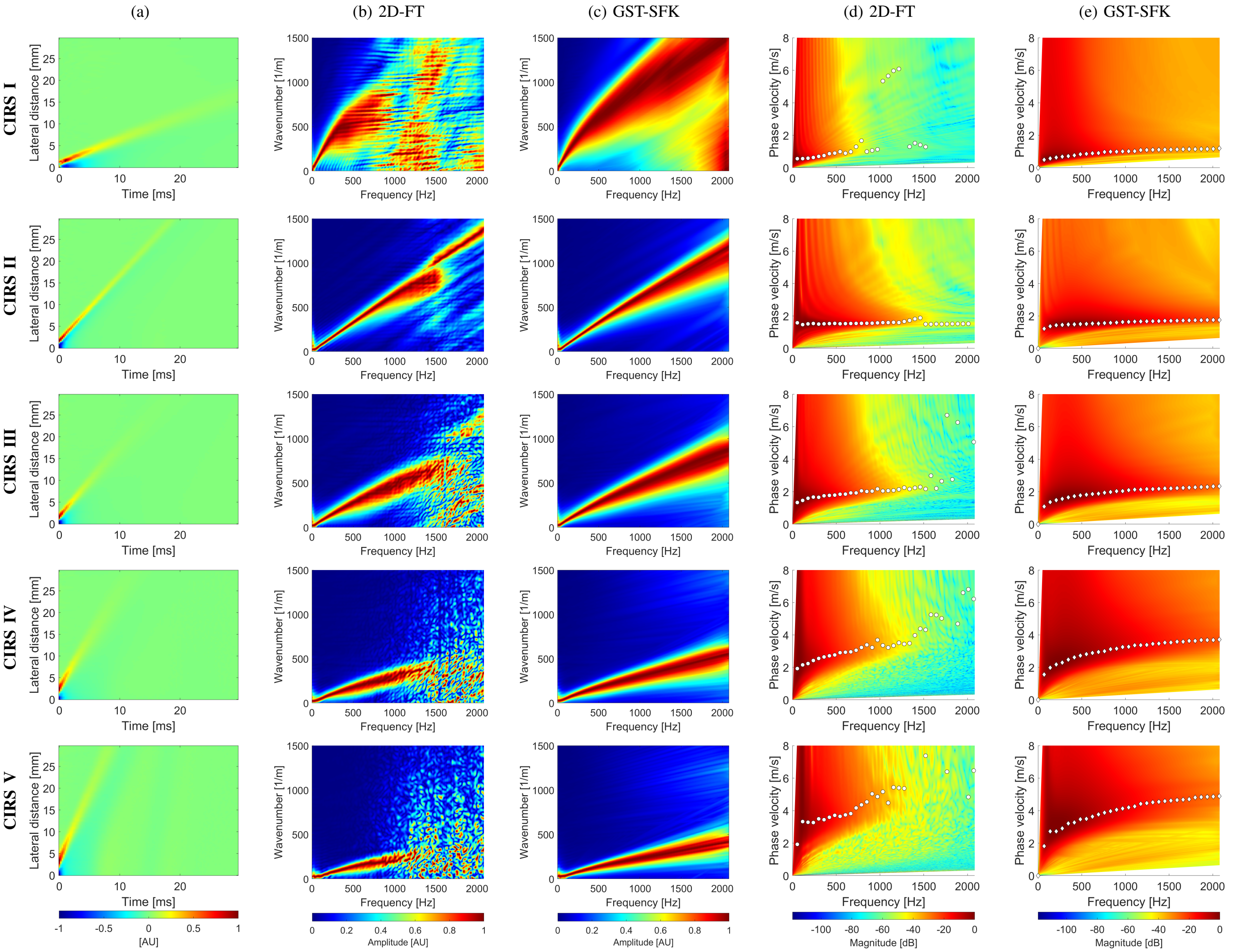

Fig. S1: (a) Spatiotemporal shear wave particle velocity signals. The frequency-wavenumber (f-k) distribution reconstructed based on the (b) 2D-FT, and (c) GST-SFK methods. The f-k maps are normalized by wavenumber maxima in the frequency direction. Phase velocity reconstructions based on the (d) 2D-FT, and (e) GST-SFK methods, for shear wave motion measurements. The phase velocity maps have superimposed markers corresponding to the maximum peaks of the phase velocity. Results were calculated for the experimental, custom-made tissue-mimicking (TM) viscoelastic phantoms I-V, for a randomly selected data acquisitions.

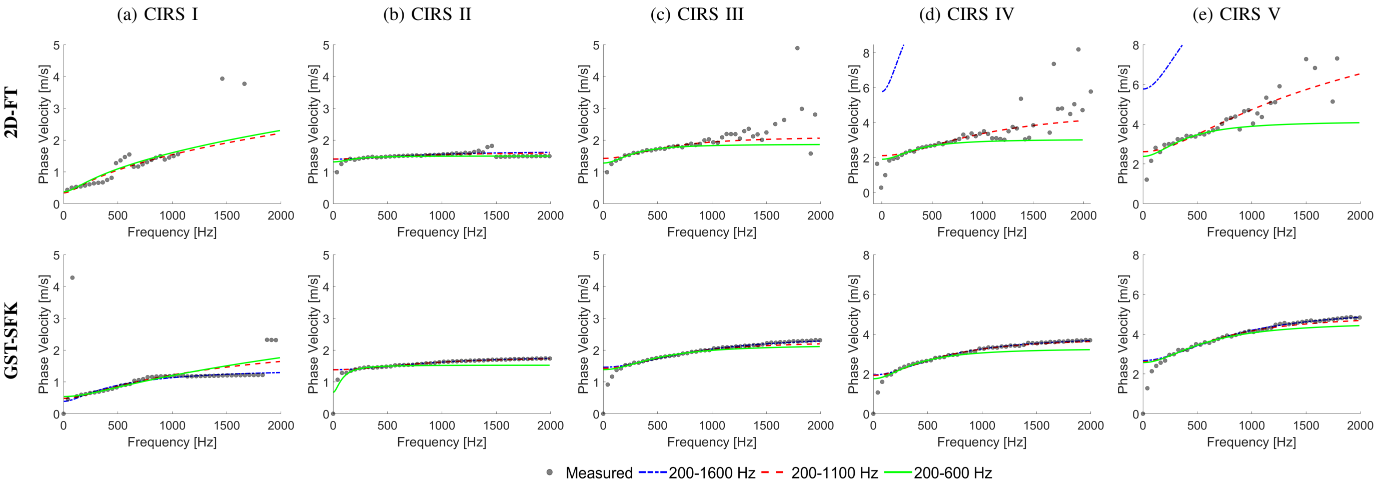

Fig. S2: Mean phase velocity dispersion curves (gray dots) measured using the 2D-FT (top row) and GST-SFK (bottom row) methods. Each figure contains fitted analytical phase velocity curves calculated using the Zener model for various frequency ranges, i.e.: 200-600 Hz, 200-1100 Hz, and 200-1600 Hz. Results were calculated for the CIRS tissue-mimicking phantoms.

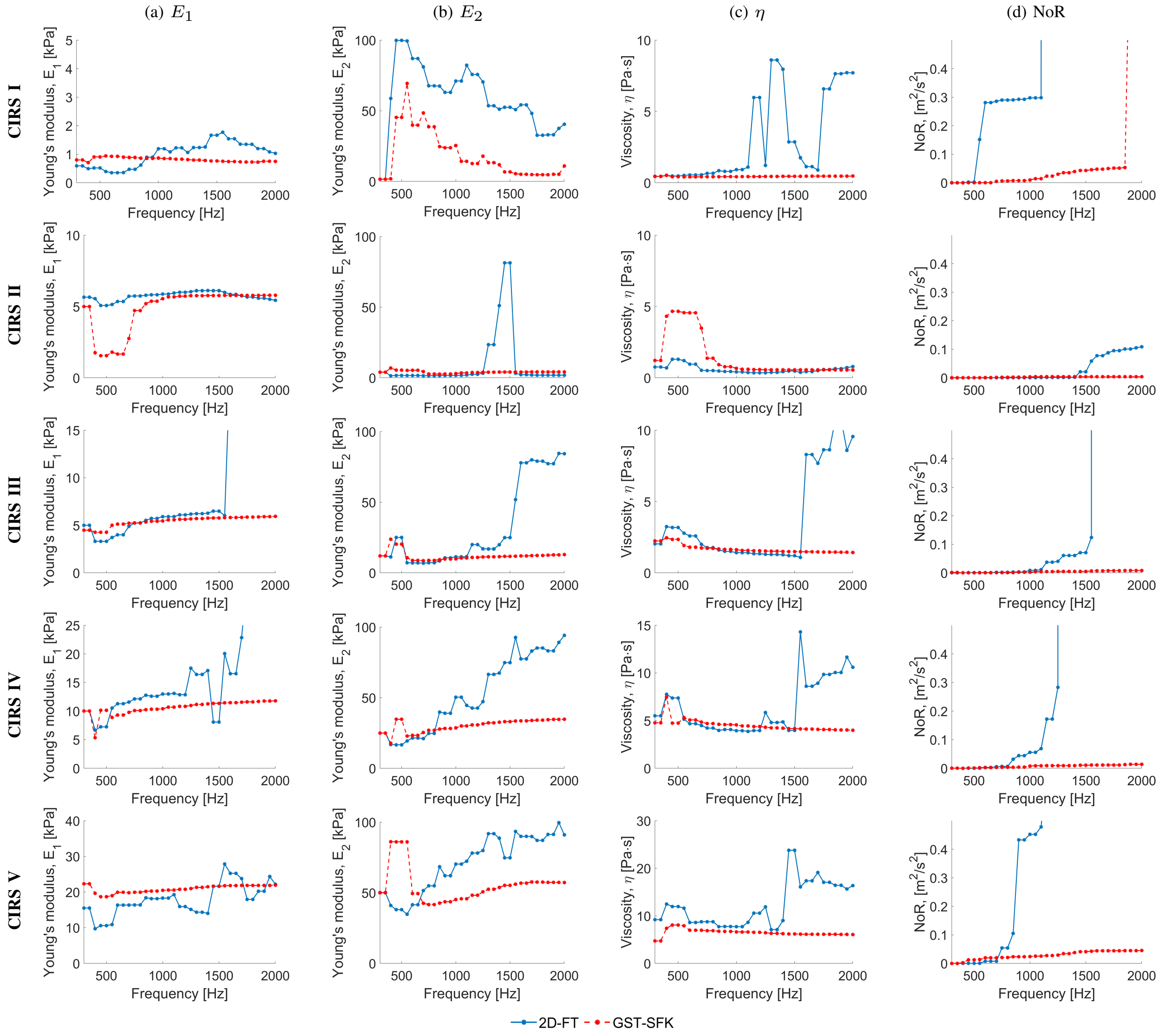

Fig. S3: Convergence analysis of the Zener model fit. Results were computed for the CIRS tissue-mimicking viscoelastic phantoms. The mean phase velocity curves obtained using the 2D-FT and GST-SFK methods were used for fitting.

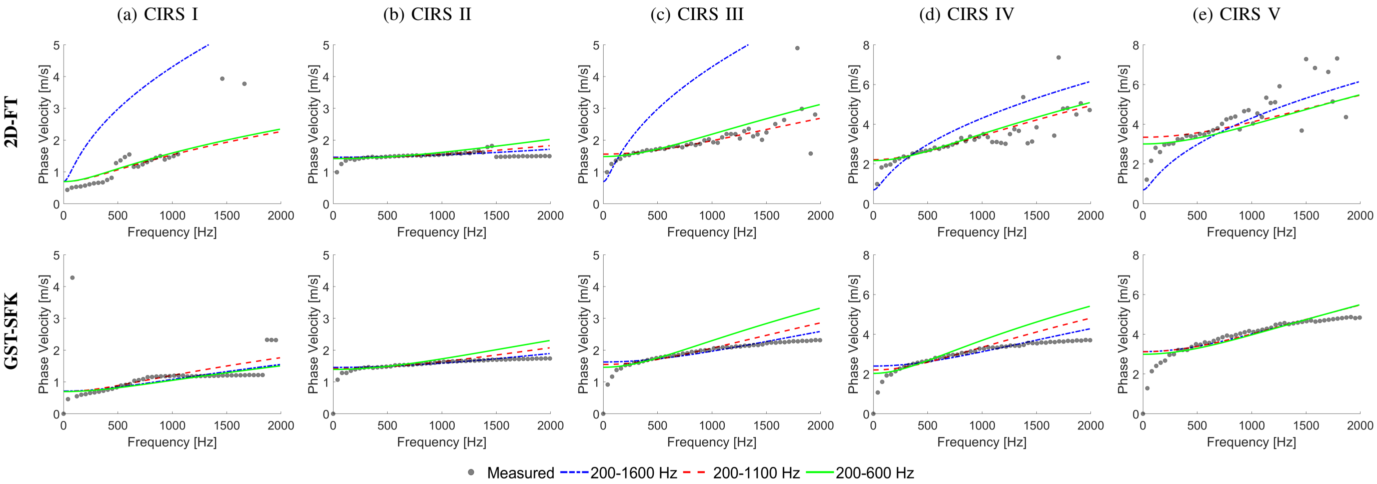

Fig. S4: Mean phase velocity dispersion curves (gray dots) measured using the 2D-FT (top row) and GST-SFK (bottom row) methods. Each figure contains fitted analytical phase velocity curves calculated using the Kelvin-Voigt model for various frequency ranges, i.e.: 200-600 Hz, 200-1100 Hz, and 200-1600 Hz. Results were calculated for the CIRS tissue-mimicking phantoms.

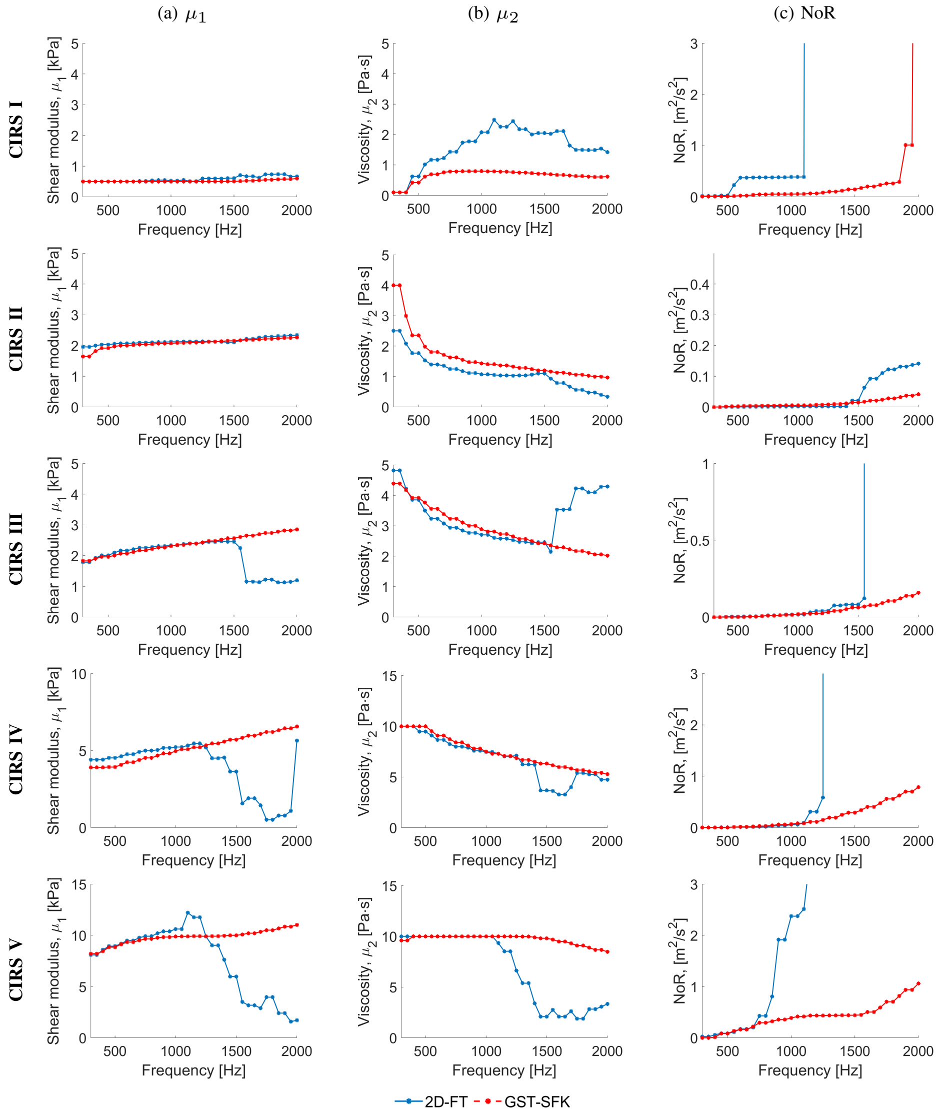

Fig. S5: Convergence analysis of the Kelvin-Voigt model fit. Results were computed for the CIRS tissue-mimicking viscoelastic phantoms. The mean phase velocity curves obtained using the 2D-FT and GST-SFK methods were used for fitting.

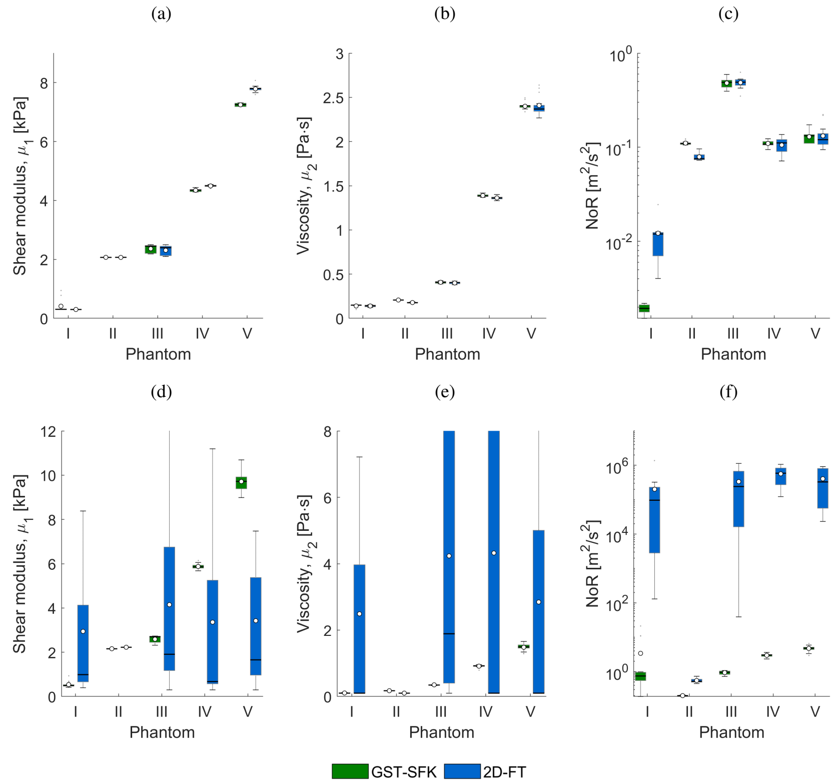

Fig. S6: Box plots calculated for estimated Kelvin-Voigt parameters (a), (d) shear modulus, μ_1, (b), (e) viscosity, μ_2, and (c), (f) the norm of residuals, NoR, for GST-SFK and 2D-FT methods. White circles represent mean values, whereas a solid line within the box corresponds to a median value. Results are presented for the TM viscoelastic phantoms I-V. The top row presents results for the KV fit performed using a short frequency ranges: 100-400 Hz for I, 100-1400 Hz for II, 100-1400 Hz for III, 150-700 Hz for IV, and 150-600 Hz for V. The bottom row shows estimates for the KV fit done using an extended frequency range from 100 to 1800 Hz for all the phantoms. Note the logarithmic vertical axis for the norm of residuals in plots (c) and (f).

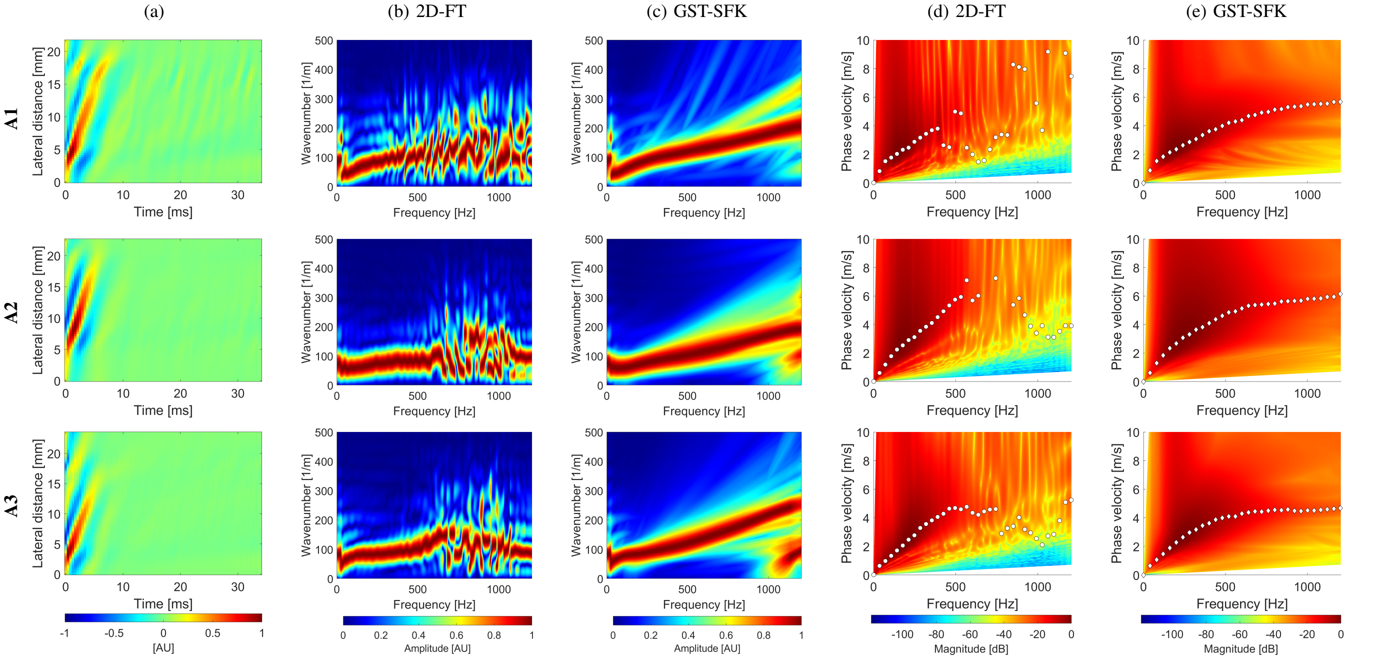

Fig. S7: (a) Spatiotemporal shear wave particle velocity signals. The frequency-wavenumber (f-k) distribution reconstructed based on the (b) 2D-FT, and (c) GST-SFK methods. The f-k maps are normalized by wavenumber maxima in the frequency direction. Phase velocity reconstructions based on the (d) 2D-FT, and (e) GST-SFK methods, for shear wave motion measurements. The phase velocity maps have superimposed markers corresponding to the maximum peaks of the phase velocity. Results were calculated for the normal (Group A) *in vivo* renal transplants, for randomly selected data acquisitions.

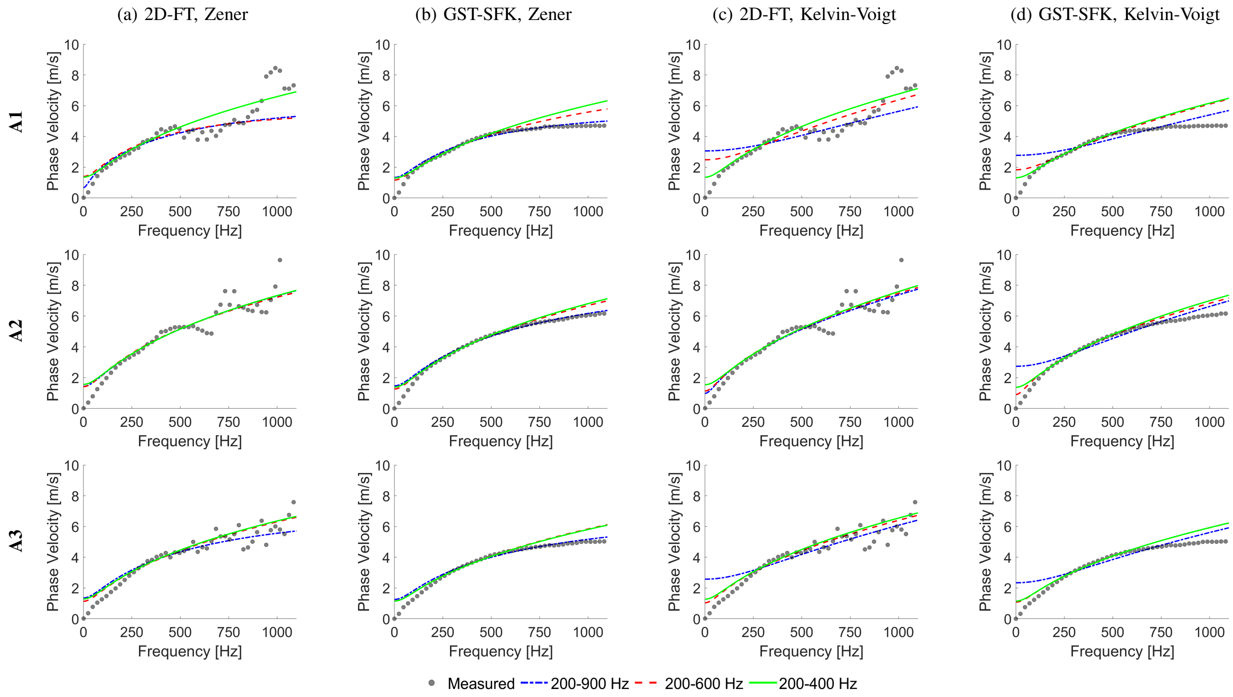

Fig. S8: Mean phase velocity dispersion curves (gray dots) measured using the (a), (c) 2D-FT and (b), (d) GST-SFK methods. Each figure contains fitted analytical phase velocity curves calculated using the Kelvin-Voigt model for various frequency ranges, i.e.: 200-400 Hz, 200-600 Hz, and 200-900 Hz. Results were computed for the *in vivo* renal transplants for the normal (Group A) *in vivo* renal transplants, for randomly selected data acquisitions.

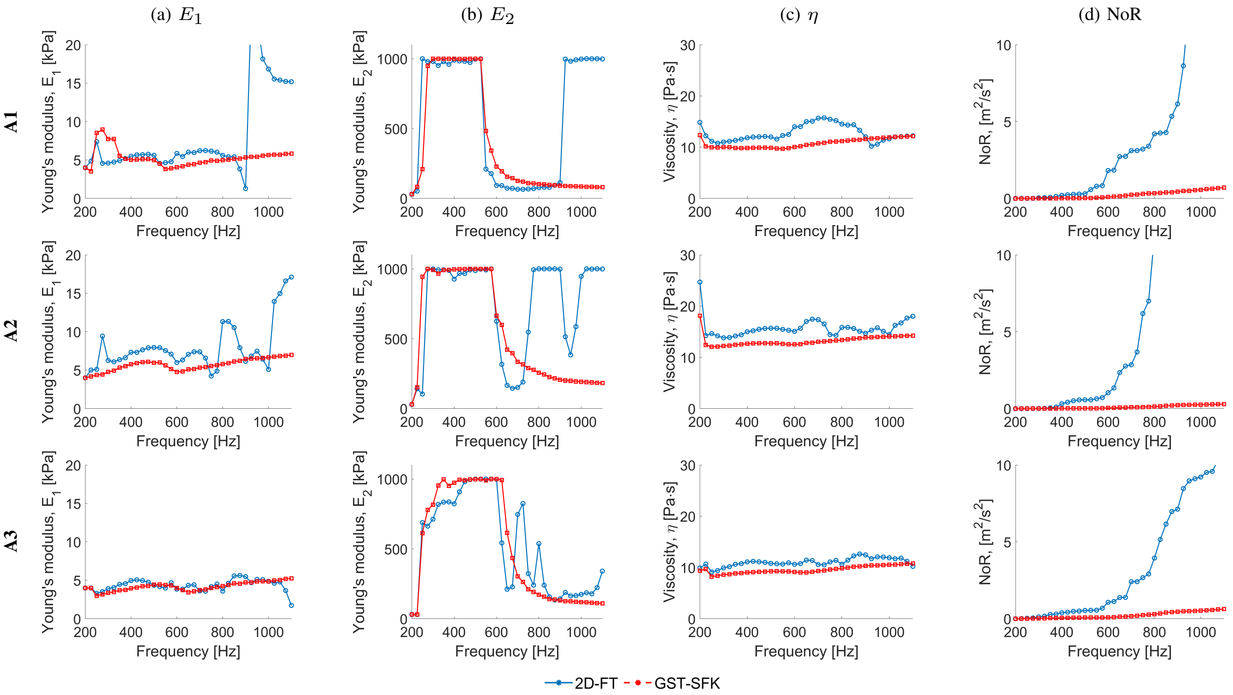

Fig. S9: Convergence analysis of the Zener model fit. Results were computed for the normal (Group A) *in vivo* renal transplants, for randomly selected data acquisitions. The mean phase velocity curves obtained using the 2D-FT and GST-SFK methods were used for fitting.

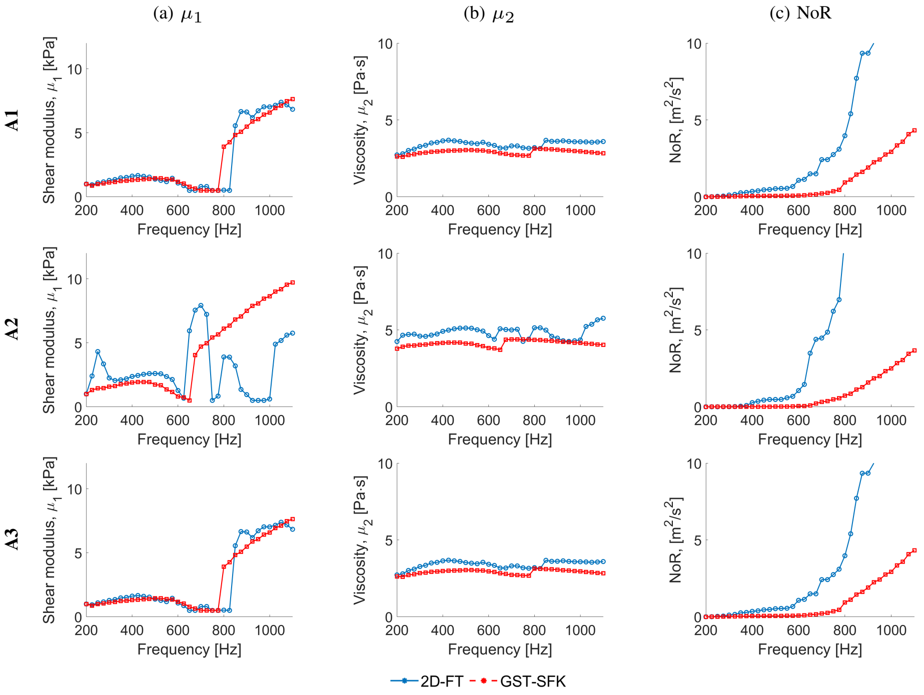

Fig. S10: Convergence analysis of the Kelvin-Voigt model fit. Results were computed for the normal (Group A) *in vivo* renal transplants, for randomly selected data acquisitions. The mean phase velocity curves obtained using the 2D-FT and GST-SFK methods were used for fitting.

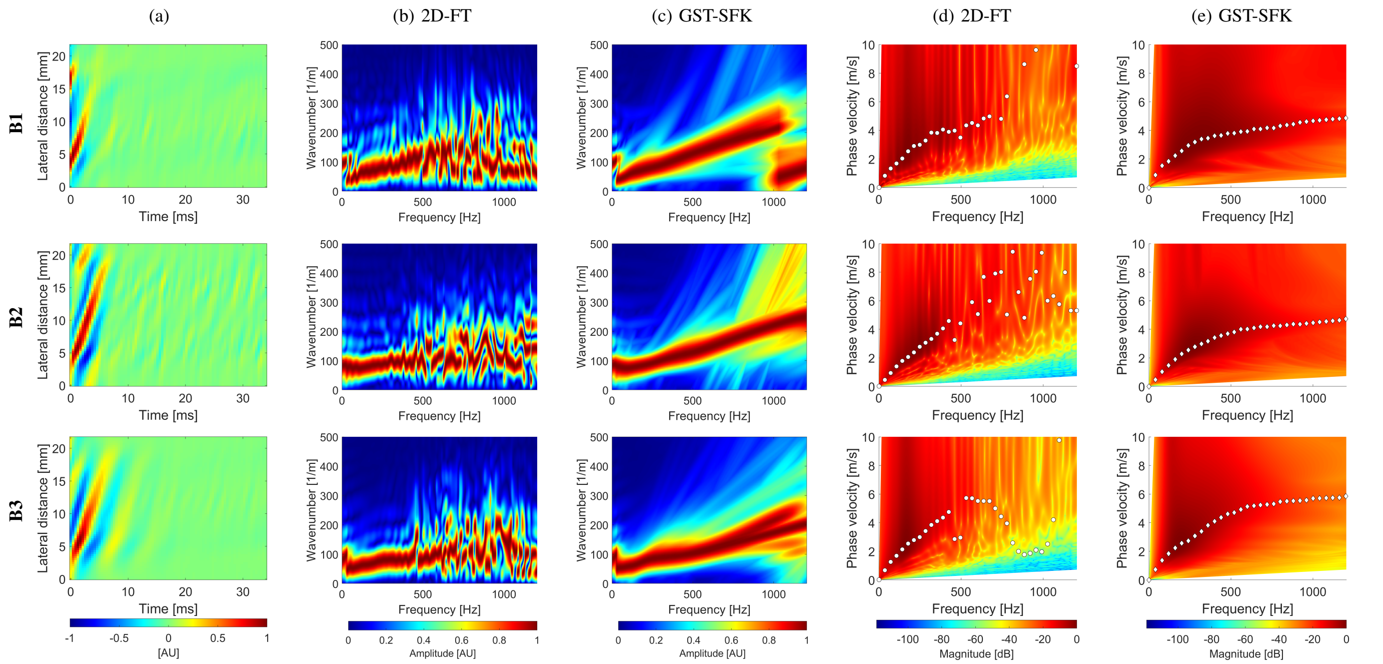

Fig. S11: (a) Spatiotemporal shear wave particle velocity signals. The frequency-wavenumber (f-k) distribution reconstructed based on the (b) 2D-FT, and (c) GST-SFK methods. The f-k maps are normalized by wavenumber maxima in the frequency direction. Phase velocity reconstructions based on the (d) 2D-FT, and (e) GST-SFK methods, for shear wave motion measurements. The phase velocity maps have superimposed markers corresponding to the maximum peaks of the phase velocity. Results were calculated for the *in vivo* renal transplants for subjects with inflammation and no IFTA (Group B), for randomly selected data acquisitions.

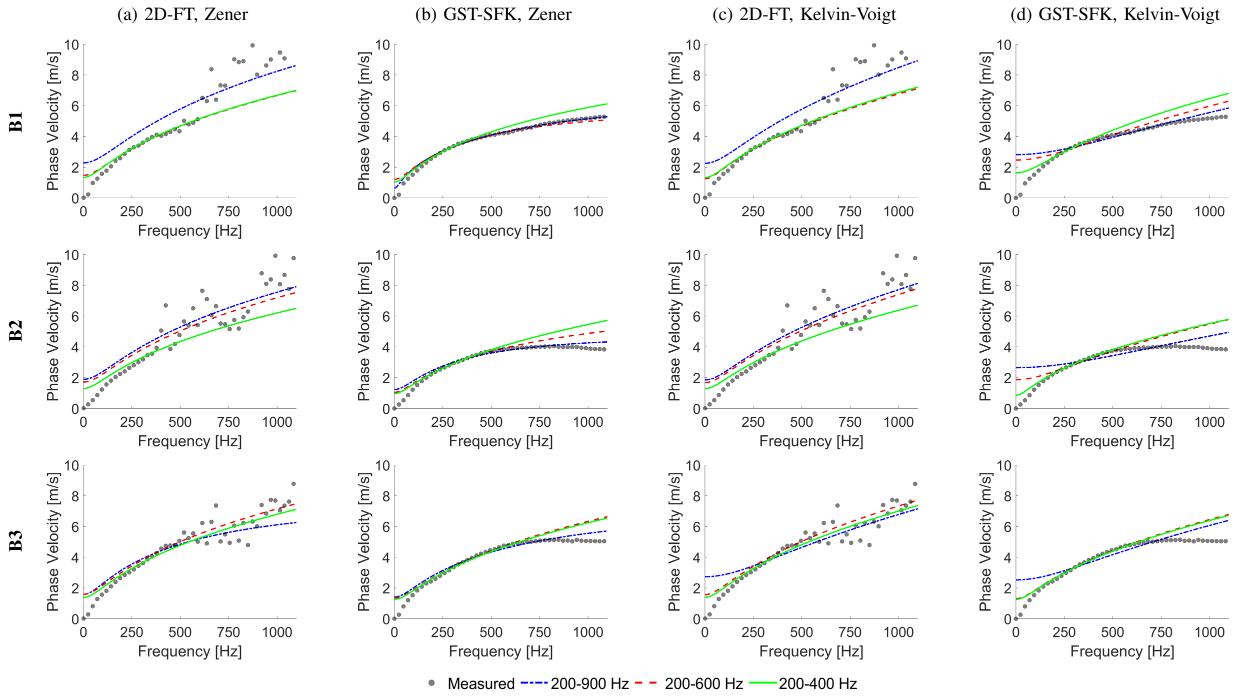

Fig. S12: Mean phase velocity dispersion curves (gray dots) measured using the (a), (c) 2D-FT and (b), (d) GST-SFK methods. Each figure contains fitted analytical phase velocity curves calculated using the Kelvin-Voigt model for various frequency ranges, i.e.: 200-400 Hz, 200-600 Hz, and 200-900 Hz. Results were computed for the *in vivo* renal transplants for subjects with inflammation and no IFTA (Group B), for randomly selected data acquisitions.

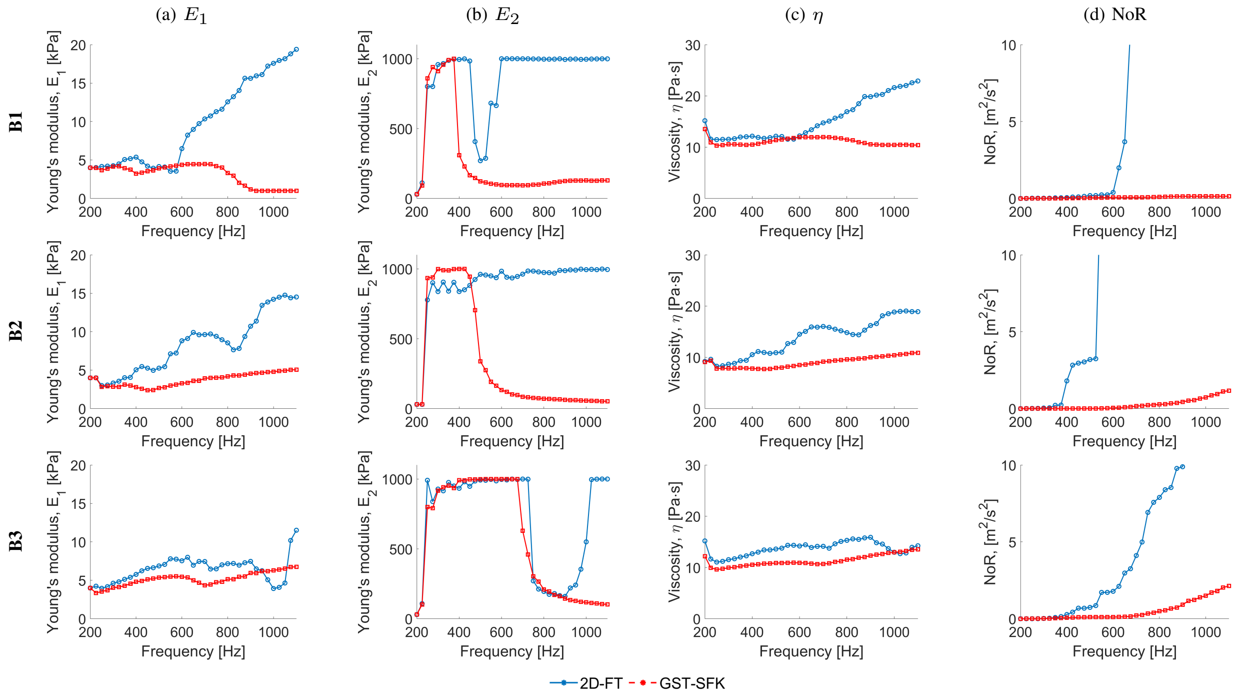

Fig. S13: Convergence analysis of the Zener model fit. Results were computed for the *in vivo* renal transplants for subjects with inflammation and no IFTA (Group B), for randomly selected data acquisitions. The mean phase velocity curves obtained using the 2D-FT and GST-SFK methods were used for fitting.

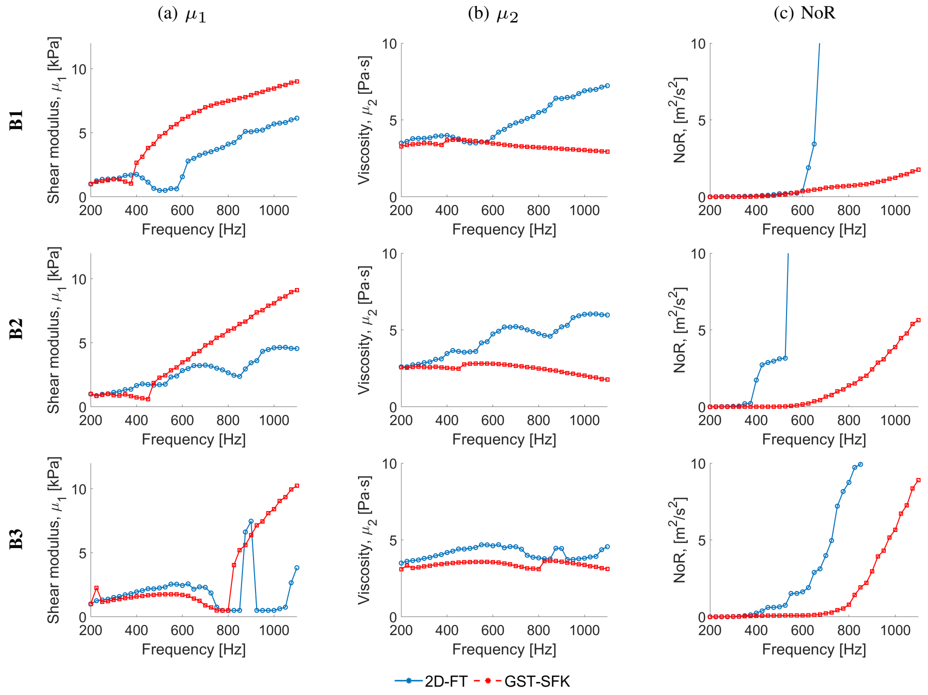

Fig. S14: Convergence analysis of the Kelvin-Voigt model fit.Results were computed for the *in vivo* renal transplants for subjects with inflammation and no IFTA (Group B), for randomly selected data acquisitions. The mean phase velocity curves obtained using the 2D-FT and GST-SFK methods were used for fitting.

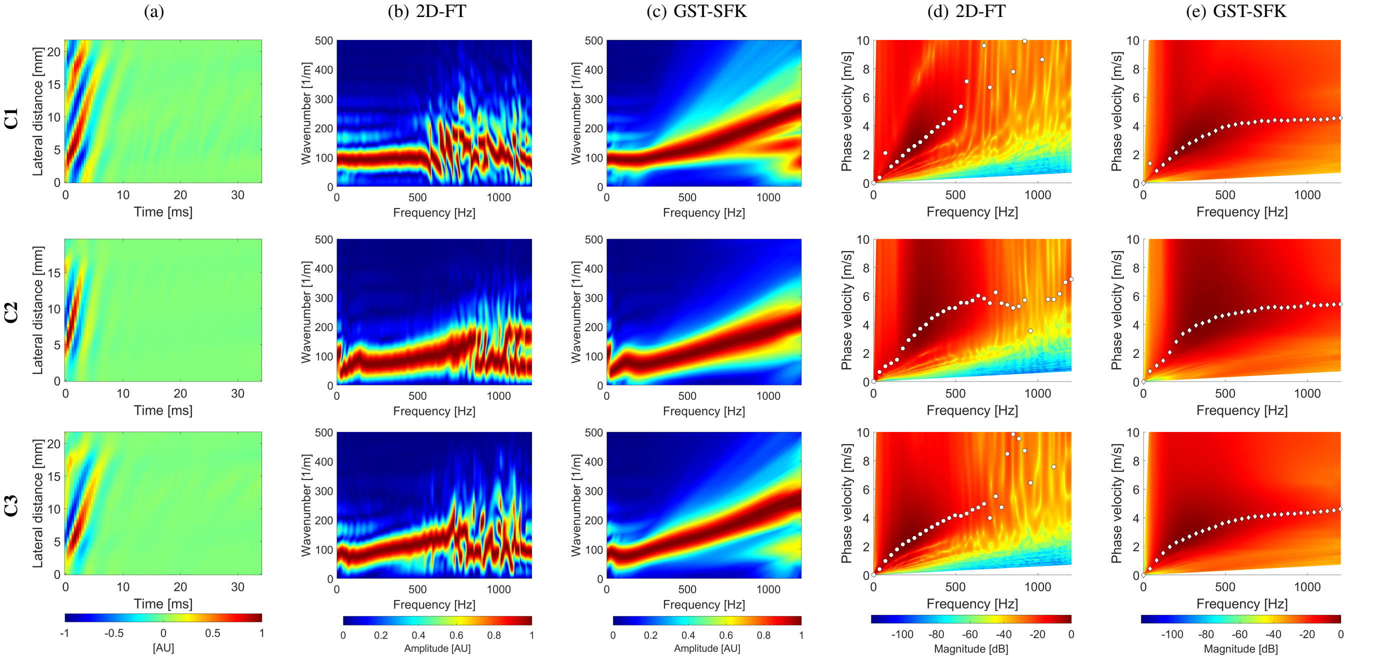

Fig. S15: (a) Spatiotemporal shear wave particle velocity signals. The frequency-wavenumber (f-k) distribution reconstructed based on the (b) 2D-FT, and (c) GST-SFK methods. The f-k maps are normalized by wavenumber maxima in the frequency direction. Phase velocity reconstructions based on the (d) 2D-FT, and (e) GST-SFK methods, for shear wave motion measurements. The phase velocity maps have superimposed markers corresponding to the maximum peaks of the phase velocity. Results were calculated for the *in vivo* renal transplants for subjects with IFTA but no inflammation (Group C), for randomly selected data acquisitions.

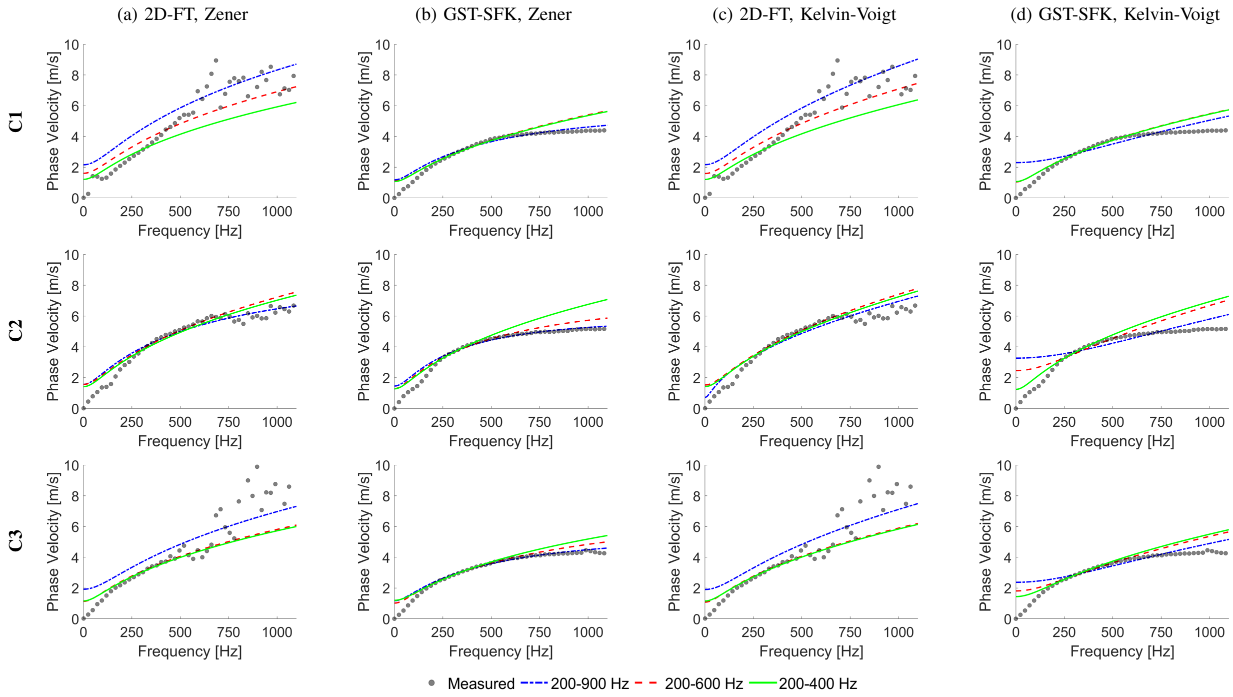

Fig. S16: Mean phase velocity dispersion curves (gray dots) measured using the (a), (c) 2D-FT and (b), (d) GST-SFK methods. Each figure contains fitted analytical phase velocity curves calculated using the Kelvin-Voigt model for various frequency ranges, i.e.: 200-400 Hz, 200-600 Hz, and 200-900 Hz. Results were computed for the *in vivo* renal transplants for subjects with IFTA but no inflammation (Group C), for randomly selected data acquisitions.

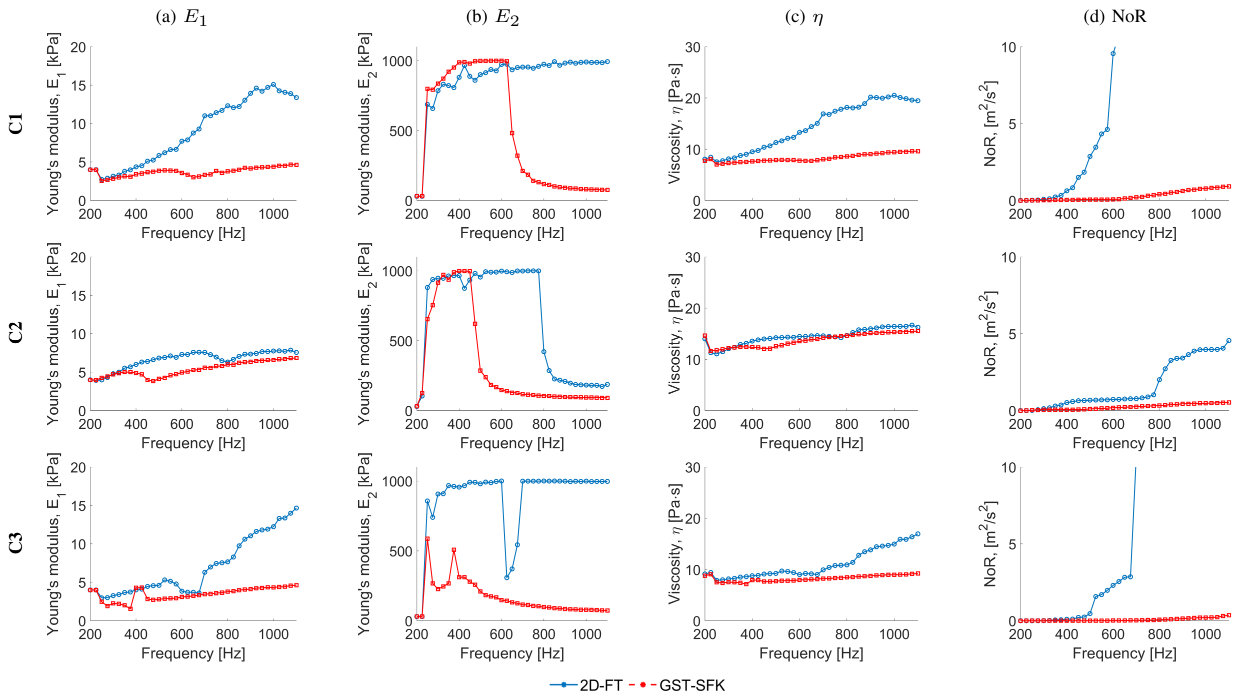

Fig. S17: Convergence analysis of the Zener model fit. Results were computed for the *in vivo* renal transplants for subjects with IFTA but no inflammation (Group C), for randomly selected data acquisitions. The mean phase velocity curves obtained using the 2D-FT and GST-SFK methods were used for fitting.

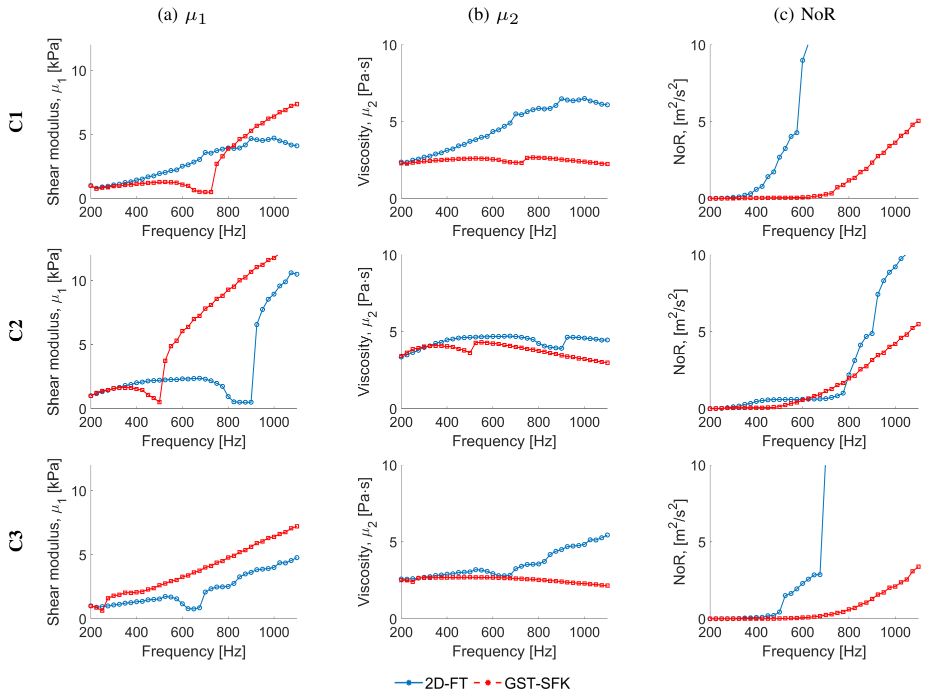

Fig. S18: Convergence analysis of the Kelvin-Voigt model fit. Results were computed for the *in vivo* renal transplants for subjects with IFTA but no inflammation (Group C), for randomly selected data acquisitions. The mean phase velocity curves obtained using the 2D-FT and GST-SFK methods were used for fitting.

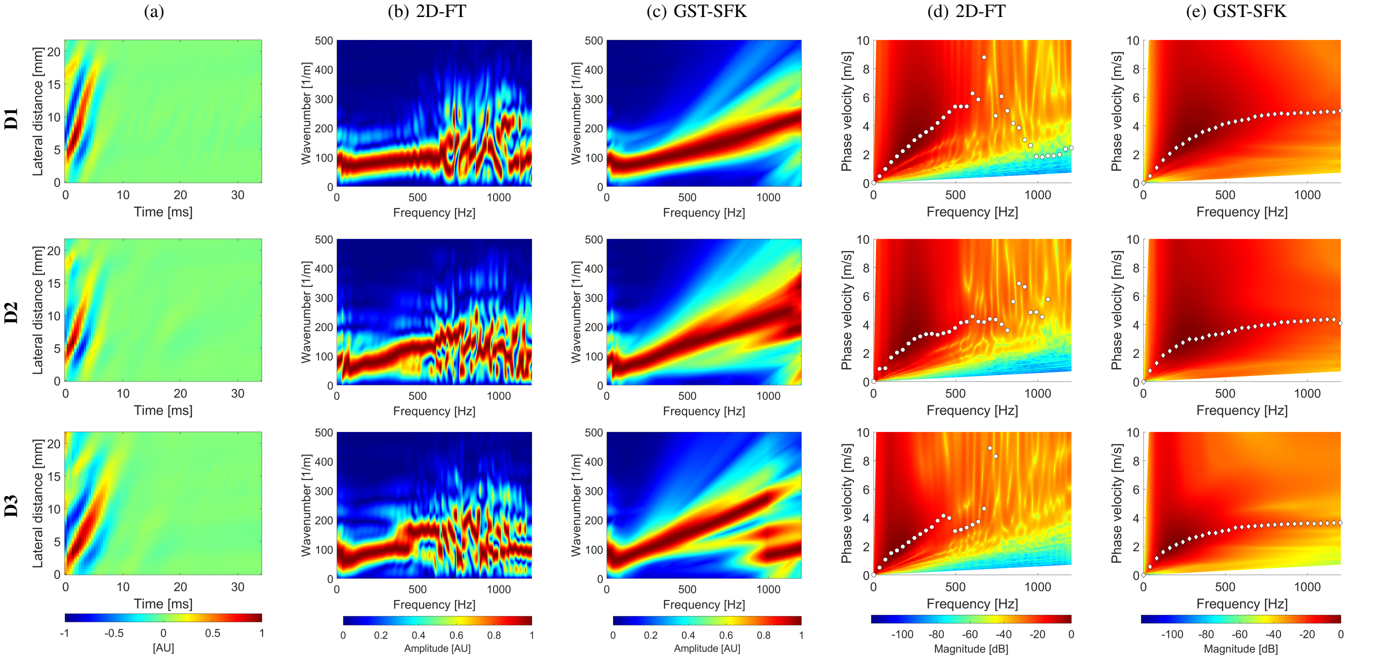

Fig. S19: (a) Spatiotemporal shear wave particle velocity signals. The frequency-wavenumber (f-k) distribution reconstructed based on the (b) 2D-FT, and (c) GST-SFK methods. The f-k maps are normalized by wavenumber maxima in the frequency direction. Phase velocity reconstructions based on the (d) 2D-FT, and (e) GST-SFK methods, for shear wave motion measurements. The phase velocity maps have superimposed markers corresponding to the maximum peaks of the phase velocity. Results were calculated for the *in vivo* renal transplants for subjects with IFTA and inflammation (Group D), for randomly selected data acquisitions.

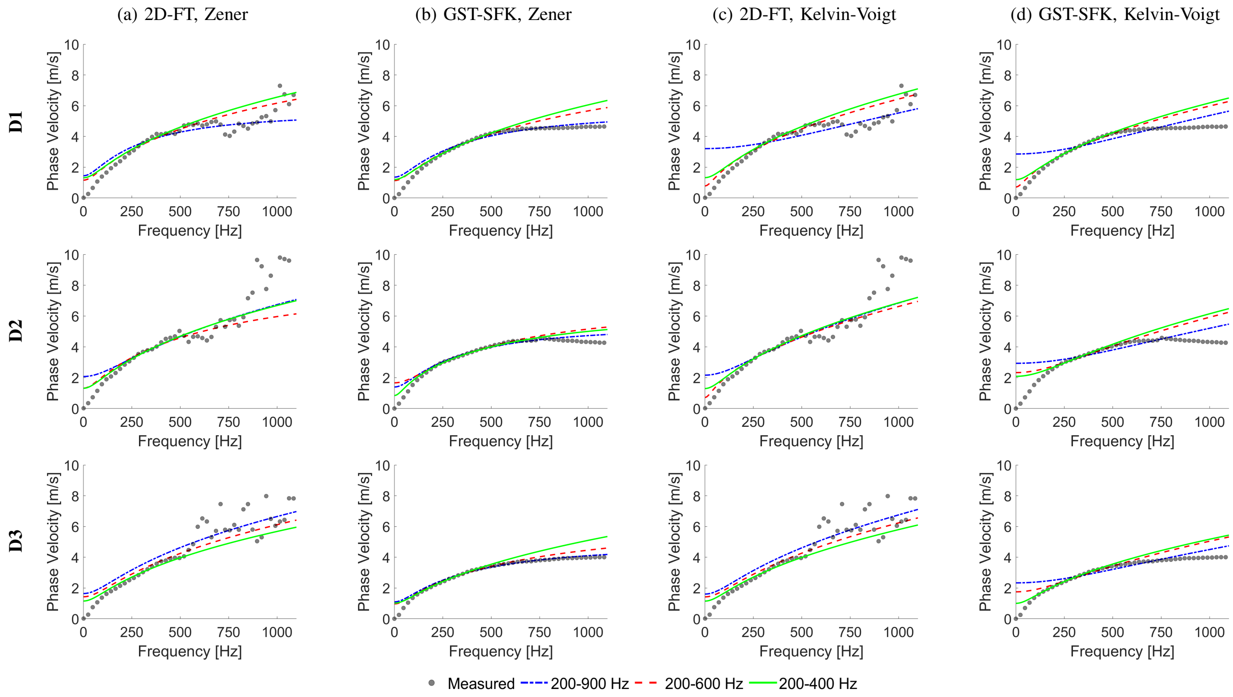

Fig. S20: Mean phase velocity dispersion curves (gray dots) measured using the (a), (c) 2D-FT and (b), (d) GST-SFK methods. Each figure contains fitted analytical phase velocity curves calculated using the Kelvin-Voigt model for various frequency ranges, i.e.: 200-400 Hz, 200-600 Hz, and 200-900 Hz. Results were computed for the *in vivo* renal transplants for subjects with IFTA and inflammation (Group D), for randomly selected data acquisitions.

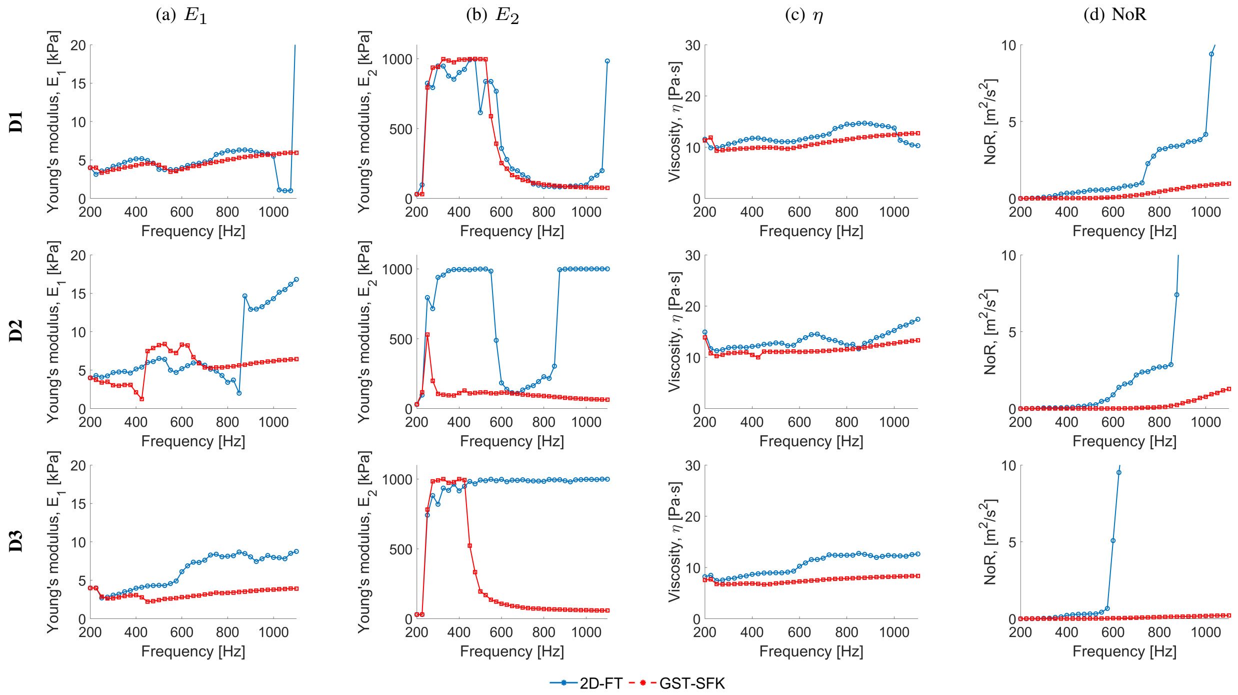

Fig. S21: Convergence analysis of the Zener model fit. Results were computed for the *in vivo* renal transplants for subjects with IFTA and inflammation (Group D), for randomly selected data acquisitions. The mean phase velocity curves obtained using the 2D-FT and GST-SFK methods were used for fitting.

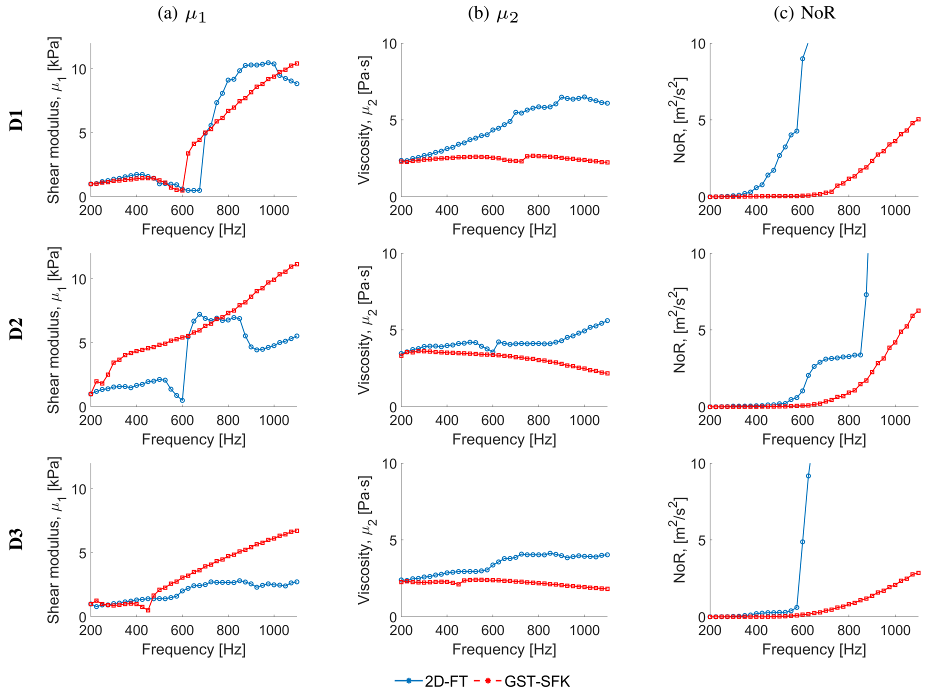

Fig. S22: Convergence analysis of the Kelvin-Voigt model fit. Results were computed for the *in vivo* renal transplants for subjects with IFTA and inflammation (Group D), for randomly selected data acquisitions. The mean phase velocity curves obtained using the 2D-FT and GST-SFK methods were used for fitting.

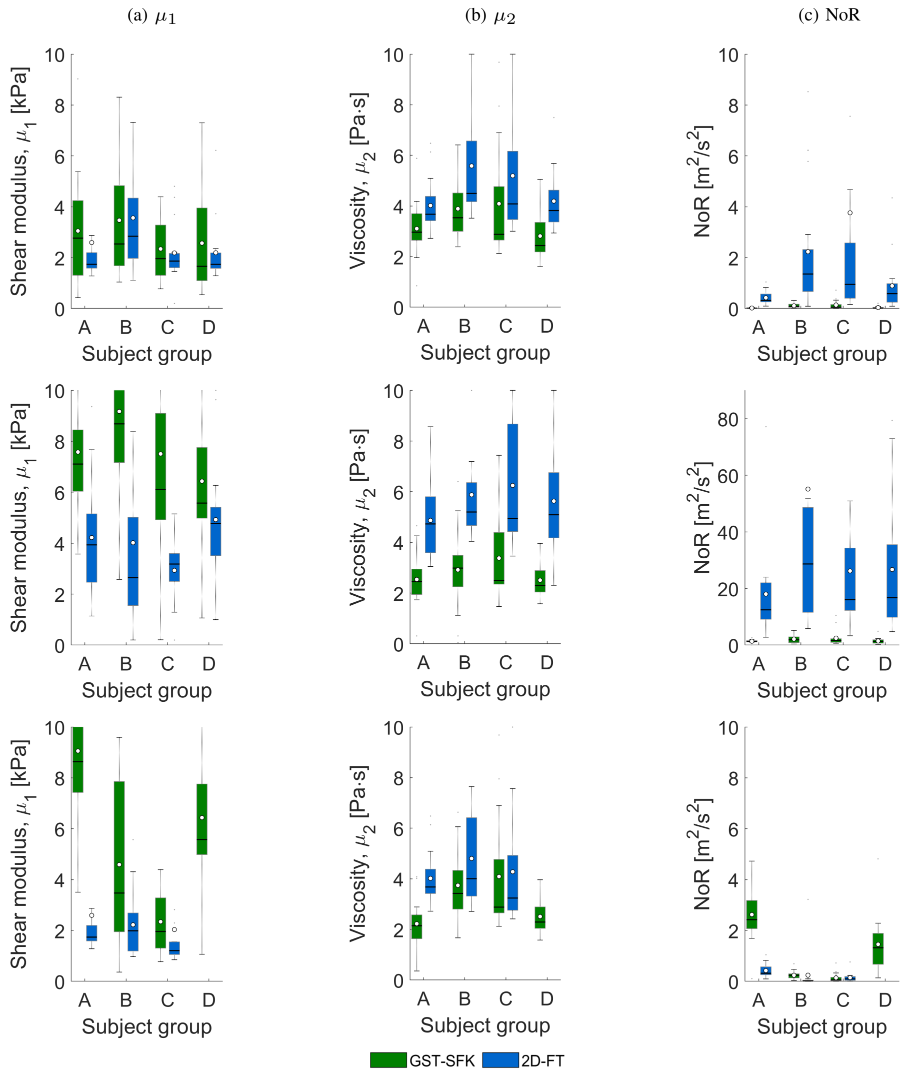

Fig. S23: Box plots calculated for estimated Kelvin-Voigt parameters (a) shear modulus, μ_1, (b) viscosity, μ_2, and (c) the norm of residuals, NoR, for GST-SFK and 2D-FT methods. White circles represent mean values, whereas a solid line within the box corresponds to a median value. A fixed frequency range of 200-450 Hz was used for the KV fit in the top row, for both techniques, where all groups (except D for 2D-FT) had CV $< 30\%$ (Case 1, top row). The middle row presents the KV fit for frequency range of 200-900 Hz (Case 2, middle row). The bottom row shows the KV fit for frequency range starting from 200 Hz up to the maximum frequency for which CV $< 30\%$ for a given subject group and given approach (Case 3, bottom row). Results are presented for the *in vivo* renal transplant data, for subject groups A-D. All groups consisted of 15 subjects each.
